# Did Photosymbiont Bleaching Lead to the Demise of Planktic Foraminifer Morozovella at the Early Eocene Climatic Optimum?

**DOI:** 10.1002/2017PA003138

**Published:** 2017-11-06

**Authors:** Valeria Luciani, Roberta D'Onofrio, Gerald R. Dickens, Bridget S. Wade

**Affiliations:** ^1^ Department of Physics and Earth Sciences Ferrara University Ferrara Italy; ^2^ Department of Earth Science Rice University Houston TX USA; ^3^ Department of Earth Sciences University College London London UK

**Keywords:** Early Eocene Climatic Optimum, planktic foraminifera, *Morozovella* decline, bleaching, carbon cycle

## Abstract

The symbiont‐bearing mixed‐layer planktic foraminiferal genera Morozovella and Acarinina were among the most important calcifiers of early Paleogene tropical–subtropical oceans. A marked and permanent switch in the abundance of these genera is known to have occurred at low‐latitude sites at the beginning of the Early Eocene Climatic Optimum (EECO), such that the relative abundance of Morozovella permanently and significantly decreased along with a progressive reduction in the number of species; concomitantly, the genus Acarinina almost doubled its abundance and diversified. Here we examine planktic foraminiferal assemblages and stable isotope compositions of their tests at Ocean Drilling Program Site 1051 (northwest Atlantic) to detail the timing of this biotic event, to document its details at the species level, and to test a potential cause: the loss of photosymbionts (bleaching). We also provide stable isotope measurements of bulk carbonate to refine the stratigraphy at Site 1051 and to determine when changes in Morozovella species composition and their test size occurred. We demonstrate that the switch in Morozovella and Acarinina abundance occurred rapidly and in coincidence with a negative carbon isotope excursion known as the J event (~53 Ma), which marks the start of the EECO. We provide evidence of photosymbiont loss after the J event from a size‐restricted δ^13^C analysis. However, such inferred bleaching was transitory and also occurred in the acarininids. The geologically rapid switch in planktic foraminiferal genera during the early Eocene was a major evolutionary change within marine biota, but loss of photosymbionts was not the primary causal mechanism.

## Key Points

- The relative abundance of the Morozovella genus permanently decreased significantly at the J carbon isotope excursion (~53 Ma)
- Changes in bulk sediment stable isotopes correlate with changes in the abundance, composition, and size of the Morozovella species
- Photosymbiont inhibition was probably not the primary cause of the remarkable Morozovella decline

## 1 Introduction

**Figure 1:**
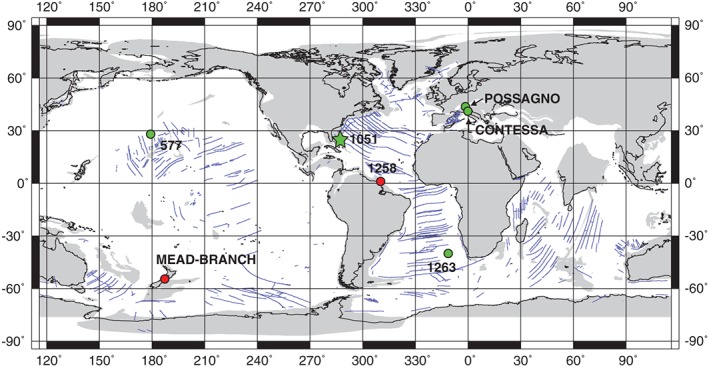
Earth at ~50 Ma showing the approximate locations of the studied site (star) and other successions (solid circles) pertinent to understanding planktic foraminiferal changes across EECO. Green circles show the localities where a permanent drop in morozovellid abundance at the EECO onset is recorded. Red circles indicate localities with early Eocene stable isotope stratigraphy. Base map is from http://www.odsn/de/services/paleomap.html with paleolatitudes modified for Sites 577, 1051, and 1263 according to www.paleolatitude.org model version 1.2 (Van Hinsbergen et al., 2015). The paleolatitudes of the Possagno, Contessa, Mead Stream and Branch Stream sections are based on the http://www.odsn.de/odsn/services/paleomap/adv_map.html model.

The symbiont‐bearing mixed‐layer genera *Morozovella* and *Acarinina* were among the most important calcifiers of early Paleogene tropical and subtropical oceans (e.g., Aze et al., 2011; Boersma et al., 1987; Pearson et al., 2006; Premoli Silva & Boersma, 1988). At multiple low‐latitude sites (Figure 1), a remarkable and permanent switch in their abundance occurred close to the start of the Early Eocene Climatic Optimum (EECO; Luciani et al., 2016). Specifically, *Morozovella* abundances decreased significantly while *Acarinina* abundances almost doubled (Luciani et al., 2016). This major turnover further relates to taxonomic diversity, consisting of species reduction among *Morozovella* and species diversification among *Acarinina* (Aze et al., 2011; Pearson et al., 2006). This turnover occurred also in the temperate Southern Hemisphere setting of Atlantic Site 1263 (Luciani et al., 2017). The timing of the *Morozovella–Acarinina* switch is interesting because the EECO (~49–53 Ma; Lauretano et al., 2015; Luciani et al., 2016; Slotnick et al., 2012, 2015) represents the interval of peak sustained Cenozoic warmth (Bijl et al., 2009; Hollis et al., 2012; Huber & Caballero, 2011; Inglis et al., 2015; Pross et al., 2012; Zachos et al., 2008). Consequently, the turnover might somehow relate to the onset of prolonged conditions of elevated temperature and significant change in carbon cycling.

The early Paleogene was punctuated by a series of geologically brief (~40–200 kyr) negative carbon isotope excursions (CIE; Cramer et al., 2003; Kennett & Stott, 1991; Littler et al., 2014; Lourens et al., 2005; Nicolo et al., 2007; Zachos et al., 2010). Most of these CIEs seem to correspond with global warming, net input of organic carbon, deep sea carbonate dissolution, and biotic turnovers (above references and Clyde et al., 2013; Coccioni et al., 2012; D'Onofrio et al., 2016; Dickens, 2011; Gingerich, 2003; Hönisch et al., 2012; Lauretano et al., 2016; Sexton et al., 2011; Thomas, 1998; Yamaguchi & Norris, 2012; Zachos et al., 2005). One of these events, known as “J,” occurred near the boundary between polarity chrons C24n.2r and C24n.3n and thus at ~53 Ma (Cramer et al., 2003; Lauretano et al., 2015; Slotnick et al., 2015) and coincident with the beginning of the EECO (Lauretano et al., 2015; Luciani et al., 2016; Slotnick et al., 2012, 2015).

Although suggesting the planktic foraminiferal turnover occurred close to the start of the EECO, Luciani et al. (2016) documented the switch at the genus level and at relatively low time resolution. Our aim here is twofold: (1) to refine the timing and nature of the foraminiferal turnover in terms of morozovellid species variance at high sample resolution and then (2) to explore loss of photosymbionts as a possible mechanism. The symbiotic relationship with photosynthetic algae is a key strategy adopted by many modern planktic forminiferal species (e.g., Bé, 1982; Bé et al., 1982; Hemleben et al., 1989). Algal photosymbiosis is crucial for life and calcification processes in most surface‐water dwelling planktic foraminifera and also provides energy, allowing the host to succeed in low‐nutrient environments (e.g., Bé, 1982; Bé et al., 1982; Hemleben et al., 1989). Loss of photosymbionts (aka “bleaching”) has been documented in many modern organisms (e.g., Addessi, 2001; Glynn, 1996; Grottoli et al., 2004; Peters, 1993; Suzuki et al., 2003), including benthic foraminifera from tropical oceans (e.g., Hallock, 2000; Williams & Hallock, 2004). The exact causes of photosymbiont bleaching can be manifold but may include elevated sea surface temperature and decreased pH (e.g., Douglas, 2003, and references therein), both of which may have occurred at the start of the EECO (e.g., Anagnostou et al., 2016; Zachos et al., 2008). Bleaching events have been suggested for large species of *Acarinina* and the genus *Morozovelloides* (morphologically and ecologically comparable with *Morozovella*) during the middle Eocene (Edgar et al., 2012; Wade et al., 2008).

For this study, we generate key records across the EECO at Ocean Drilling Program (ODP) Site 1051 in the northwest Atlantic. This site has a solid bio‐magneto stratigraphy and is one of the sites (Figure 1) where the morozovellid decline has been documented (Luciani et al., 2016). Specifically, we provide stable carbon and oxygen isotope curves of bulk carbonate to determine a detailed stratigraphy and evidence for CIEs and inferred increased temperatures.

We then document high‐resolution changes in the abundance of primary planktic foraminiferal genera and also in the morozovellid species and correlate these changes with the carbon isotope excursions. For selected samples, we further record variations in morozovellid maximum test size. This is because endosymbiosis relates to calcification and foraminiferal longevity, so the loss of photosymbionts should reduce the abundance and average test size of affected foraminifera (e.g., Bé et al., 1982; Caron et al., 1982; Edgar et al., 2012; Wade et al., 2008). We estimate the stable isotope composition of different test sizes to determine foraminiferal ecology and because this analysis a powerful indicator of photosymbiont activity (e.g., Spero & DeNiro, 1987). We show here that the planktic foraminiferal turnover precisely corresponds to the J event and involves major abundance declines in multiple morozovellid species. However, we suggest that the reduction in photosymbiont activity was not the primary causal mechanism for the striking long‐term switch in planktic foraminiferal evolution that marks the start of the EECO.

## 2 ODP Site 1051, Northwest Atlantic

The section selected for this study comes from ODP Site 1051 on Blake Nose in the northwest Atlantic. Site 1051, which comprises two holes (A and B), is located at 30**°**03.2**′**N, 76**°**21.5**′**W and ~1980 m water depth (Norris et al., 1998). However, during the early Eocene, the position was slightly to the south (Figure 1; Ogg & Bardot, 2001; Van Hinsbergen et al., 2015). Multiple characteristics make the lower Eocene sedimentary record at Site 1051 appropriate for understanding the major switch in planktic foraminifera. The succession predominantly consists of “siliceous nannofossil chalk,” with the siliceous component including radiolarians, diatoms, and sponge spicules (Shipboard Scientific Party, 1998). Calcareous plankton biostratigraphy (Luciani & Giusberti, 2014; Mita, 2001; Norris et al., 1998) and polarity chrons, once recalibrated (Cramer et al., 2003; Luciani et al., 2016), indicate a ~100 m depth interval spanning from ~53.5 to ~47.5 Ma according to the Global Polarity Time Scale 2012. This implies a modest sedimentation rate (>1.6 cm/kyr) for much of the early Eocene. Moreover, sediment recovery was very good except for the depth interval between 382 and 390 m below sea floor (mbsf), which contains significant chert and a hiatus (Cramer et al., 2003; Luciani et al., 2016; Shipboard Scientific Party, 1998). Initial stable isotope work on the lowermost Eocene interval (Cramer et al., 2003) also indicates that bulk sediment gives an interpretable δ^13^C record, and the J event is at ~428 mbsf.

Planktic foraminifera exhibit a “frosty” preservation (sensu Sexton et al., 2006) that implies some recrystallization, and they may have infilled tests. The latter feature is commonly observed for the species *Morozovella lensiformis* and M *orozovella* aequa. Notwithstanding, species are readily recognizable throughout the studied interval (Luciani et al., 2016; Norris et al., 1998).

We focused on the depth interval between 452.3 and 353.1 mbsf at Hole 1051A, the same as studied by Luciani et al. (2016). Within this interval, we analyzed a total of 227 samples that were nominally 20–30 cm^3^ in volume. Samples were used for various analyses, at different spacing, as specified in paragraphs below.

## 3 Methods

### 3.1 Stable Isotope Measurements

**Figure 2:**
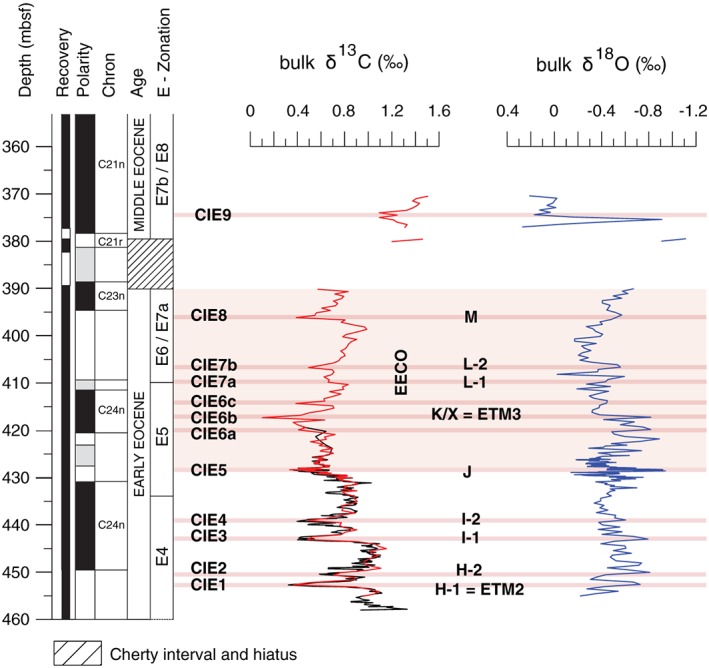
Early to early middle Eocene bulk sediment carbon and oxygen stable isotope data plotted against depth for the early Eocene interval at Ocean Drilling Program (ODP) Site 1051. The black δ^13^C curve shows data generated by Cramer et al. (2003); the red curve is from this study. Plankic foraminiferal zones were assigned by Luciani et al. (2016) following the zonation scheme presented by Wade et al. (2011), but as modified by Luciani and Giusberti (2014). Magnetostratigraphy comes from Ogg and Bardot (2001), but with an important modification to polarity chron labeling as discussed in subsequent literature (Cramer et al., 2003; Luciani et al., 2016). The thin light‐red bands highlight significant negative carbon isotope excursions (CIEs) with nomenclature following Cramer et al. (2003) and Lauretano et al. (2015). The pink shaded band defines the Early Eocene Climatic Optimum (EECO) interval, although the termination lies within the depth interval of missing core and chert.

New bulk carbon and oxygen stable isotope data were generated on 227 samples (Figure 2). The sampling resolution is 40 cm for the lower (459.26–430.78 mbsf) and upper (425.18–369.9 mbsf) intervals but between 5 cm and 20 cm across the J event (Table S1 in the supporting information). Samples were first freeze‐dried and then pulverized manually with a mortar and pestle. Powdered portions of **~**0.5 g were acidified at 50°C. Bulk isotope analyses for the lower and upper intervals were performed at the University College of London (UCL) Bloomsbury Environmental Isotope Facility using a Gas Bench II device or at Cardiff University using a Thermo Finnigan MAT 252 mass spectrometer coupled with a Kiel III carbonate preparation device. Analytical precision for both instruments was within 0.04% and 0.08‰ for δ^13^C and δ^18^O, respectively. The higher resolution samples across the J event were analyzed at Padua University with a Thermo Scientific Delta V Advantage Isotope Ratio Mass Spectrometer coupled with a Gas Bench II automated preparation device. A total of 11 duplicate analyses were conducted on selected samples giving reproducibility better than 0.1‰ and 0.2‰ for δ^13^C and δ^18^O, respectively. Results are reported in conventional delta notation (δ^13^C and δ^18^O) with reference to the Vienna Pee Dee Belemnite standard. As stable isotope records across the three laboratories align, we consider laboratory offsets as minimal.

**Figure 3:**
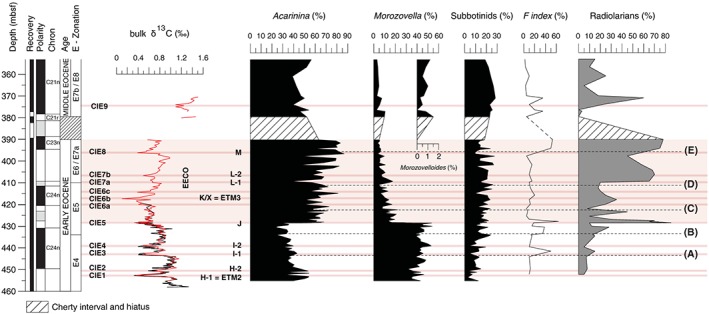
Stratigraphy, bulk sediment δ^13^C composition, fragmentation index (*F* index, from Luciani et al., 2016), and relative abundances of primary planktic foraminiferal genera and radiolarians for the early and early middle Eocene interval at ODP Site 1051 (Blake Nose). Subbotinids include the ecologically similar genera, thermocline dwellers, *Subbotina* and *Parasubbotina*. Note that the major switch in the mixed‐layer dwellers *Morozovella* and *Acarinina* abundances precisely coincides with CIE‐5, which corresponds to the J event and the onset of the EECO. (A) through (E) indicate the position of the five samples used to examine the size and stable isotope composition of *Morozovella* species. (A) and (B) are from the pre EECO interval, (C) is at the beginning of EECO, just after the J event, and (D) and (E) are from within the main EECO phase. Dashed lines help to identify the position of these samples, and other information is consistent with Figure 2.

Stable isotopes also were measured on 111 size‐constrained planktic foraminiferal samples separated from five sediment horizons specifically selected to span the onset of the EECO. The horizons are alphabetically ordered from lower to higher depth below the seafloor: A (443.58 mbsf), B (433.5 mbsf), C (425.4 mbsf), D (411.8 mbsf), and E (395.65 mbsf; Figure 3, Table S1). Analyses were conducted at Padua University as noted above.

### 3.2 Tetraether Lipid Analysis

Because significantly elevated sea‐surface temperatures (SSTs) have been linked to photosymbiont bleaching (e.g., Douglas, 2003; Edgar et al., 2012), it would be ideal to have quantitative SSTs across the studied interval. The TEX_86_ proxy has been developed and used extensively over the last decade to reconstruct Cenozoic paleotemperatures (e.g., Bijl et al., 2013; Hollis et al., 2012; Keating‐Bitonti et al., 2011; Liu et al., 2009). The approach has been explained in multiple papers (e.g., Boyd et al., 2011; Schouten et al., 2002, 2013; Uda et al., 2001). We attempted to generate TEX_86_ and BIT values as an index of terrestrial influx (Hopmans et al., 2004; Schouten et al., 2013) for the studied interval. This was largely unsuccessful, because isoprenoidal glycerol dibiphytanyl glycerol tetraethers (GDGTs) containing cyclopentane rings are very scarce throughout the studied interval. Details on methods (De Jonge et al., 2014; Schouten et al., 2002, 2013) and results are given in the supporting information.

### 3.3 Foraminiferal Analyses

#### 3.3.1 Samples and Biostratigraphy

Planktic foraminifera were studied on washed residues using a stereomicroscope with an incident light beam. The residues were prepared by immersing previously freeze‐dried samples in deionized water. When disaggregated, samples were washed over a >63 μm sieve. Washed residues were dried at <50**°**C. The taxonomic criteria adopted in this study follow Olsson et al. (1999) and Pearson et al. (2006). Planktic foraminiferal biostratigraphy comes from Luciani et al. (2016), who applied the zonal scheme of Wade et al. (2011) but included a modification (Luciani & Giusberti, 2014). Specifically, Zones E6 and E7a have to be combined at Site 1051 because the first appearance of *Acarinina cuneicamerata* is diachronous and the alternative marker species, *Astrorotalia palmerae*, is absent.

#### 3.3.2 Planktic Foraminiferal, Radiolarian Abundances, and *F* Index

Relative abundances of planktic foraminiferal genera, *Morozovella* species, and radiolarians were determined for the >63 μm size fraction from random splits using a Micro Riffle Splitter Gilson SP‐171X. However, the sample resolution varied.

Sixty‐two samples (Table S2) were analyzed to refine records at Site 1051 for the relative abundances of main planktic foraminiferal genera (i.e., *Morozovella*, *Acarinina*, and subbotinids). Relative abundances of genera were obtained by counting within a population of about 300 specimens of planktic foraminifera. The primary aims were (1) to verify the suggested temporal link between the planktic foraminiferal switch and the J event and (2) to establish whether abundance changes occurred across other early Eocene CIEs. Ten samples were collected across the J event at spacing between 5 cm and 20 cm, while the remaining samples were obtained with spacing from 40 cm to 100 cm (Table S2). Within the subbotinids are included representatives of both *Subbotina* and *Parasubbotina*, as these genera are suspected of having strong paleoecological affinities as thermocline dwellers (Pearson et al., 2006, and references therein).

Quantitative data on various *Morozovella* species were not presented in the work by Luciani et al. (2016), but this aspect may be important for understanding the early Eocene morozovellid decline and the possible cause for the planktic foraminiferal shift. Of the total samples, we selected 50 across the succession to document variations in the relative abundances of different *Morozovella* species (Table S3). The abundance of the *Morozovella* species was determined as the number of each species within a population of about 300 planktic foraminifera.

Radiolarian abundances were determined as a proxy for surface water eutrophication since they are commonly interpreted as eutrophic forms (Hallock, 1987). Their abundance was evaluated on the same 50 samples used for the *Morozovella* species analysis as the number of radiolarians with respect to planktic foraminifera and expressed in percentage as *R* = *R*/(*R* + *F*_planktic_) × 100.

Considering that deep sea carbonate dissolution, commonly associated with early Eocene negative CIEs, causes planktic foraminifera to break into fragments when they begin to dissolve (e.g, Berger, 1970; Hancock and Dickens, 2005; Nguyen and Speijer, 2014), we adopt the fragmentation index (*F* index) from Luciani et al. (2016). This proxy (expressed as a percentage) has been calculated according to Berger (1970): the ratio between fragments or partially dissolved planktic foraminiferal tests versus entire tests on ~300 elements. The fragmented tests include all planktic foraminiferal specimens showing missing or deteriorated chambers and substantial breakage.

#### 3.3.3 Maximum Size of *Morozovella* Species and *Acarinina*

To examine variations in morozovellid test size across the EECO, we measured the maximum linear length perpendicular to the coiling axis of all specimens present in the >300 μm size fraction (generally 100 specimens) of the five alphabetically labeled samples (Figure 3). Measurements were taken using the stereomicroscope at 80X magnification using the Zeiss ZEN‐Core software with instrumental error of ±1 μm (Table S4). We calculated, for each of the five samples, the mean value and the relative standard deviation (1*σ*) of test size for each morphologically defined morozovellid species. We provide also measures of the maximum test size for the genus *Acarinina* counting all the specimens occurring in the >300 μm size fraction (Table S4).

#### 3.3.4 Size‐Restricted δ^13^C Analyses

Algal symbionts preferentially remove the lighter ^12^C isotope during photosynthesis, leaving adjacent water enriched in ^13^C. A characteristic increase in δ^13^C with increasing test size thus occurs in tests of modern and fossil species because larger specimens support greater dinoflagellate symbiont density and enhanced photosynthetic activity (e.g., Spero & DeNiro, 1987). In fact, such a relationship has provided supporting evidence of photosymbiotic activity in *Morozovella* and *Acarinina* (e.g., Norris, 1998; Spero & DeNiro, 1987). In short, even though photosymbionts are not preserved in the fossil record, indirect evidence for their presence in planktic foraminifera of the past can be deciphered through the stable isotope composition of differently sized specimens (e.g., Birch et al., 2012; Bornemann & Norris, 2007; D'Hondt et al., 1994; Elderfield et al., 2002; Norris, 1996; Pearson et al., 1993; Quillévéré et al., 2001; Takagi et al., 2015; Wade et al., 2008; Wendler et al., 2013).

**Figure 4:**
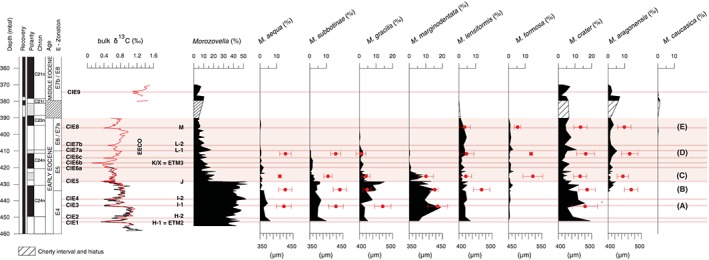
The bulk sediment δ^13^C curve across the early and early middle Eocene interval at ODP Site 1051 along with relative abundances of the *Morozovella* species. Note that the major permanent decline in *Morozovella* at the EECO onset mainly happens because of reductions in M. gracilis and *M. marginodentata* abundances and, to a lesser extent, *M. crater* and *M. subbotinae* abundances. Red symbols for each *Morozovella* species indicate the mean values (solid circle) and standard deviation (bar) for the size of tests present in the ≥300 μm fraction (generally ~100). Note the drop in test size for almost all *Morozovella* species in sample C. The thin light‐red bands highlight the main carbon isotope excursions (CIEs). Other information is the same as in previous figures.

As bleaching might affect stable carbon isotope composition (e.g., Bé et al., 1982; Caron et al., 1982; Edgar et al., 2012; Wade et al., 2008; Wade & Olsson, 2009), we generated δ^13^C data for planktic foraminiferal specimens of different sizes. The five samples noted in section 3.1 (A–E) selected for this exercise are the same five noted above (Figures 3 and 4). The species examined were *Morozovella aragonensis*, *Morozovella crater*, *Morozovella* gracilis, *Morozovella marginodentata*, *and Morozovella subbotinae*. We also examined samples of *Acarinina* spp. and of the asymbiotic genus *Subbotina* (e.g., Pearson et al., 2006, and reference therein). In order to limit interspecific variability, we mainly focused on *Acarinina esnaensis* and *Acarinina* interposita in samples A, B, and C, and on *Acarinina quetra* in samples D and E, because they are the most common and best preserved forms in the respective samples. For the subbotinids, analysis mainly included samples of *Subbotina* patagonica and *Subbotina roesnaensis*.

Specimens were picked from restricted size fractions, separated by dry sieving: 150–200 μm, 200–250 μm, 250–300 μm, 300–350 μm, and 350–400 μm. In order to generate reproducible δ^13^C records, the picked specimens were carefully checked for preservation, and heavily recrystallized or infilled tests were removed, as they would have isotope signals heavily modified by diagenesis (e.g., Pearson, 2012; Pearson et al., 2001; Sexton et al., 2006). Between 10 and 50 individuals, depending on availability, were analyzed from each size fraction to reach at least 250 μg. Notably, while morozovellids were present in the >350 μm fraction, they were generally too scarce or too poorly preserved for selecting a quantity amenable for our stable isotope analysis. We were not able to analyze *Morozovella formosa* in such a manner, as its scarce abundance throughout did not allow for sufficient specimens in the different size fractions. The scarcity of *M. marginodentata* in sample D also did not permit collection of an adequate number of specimens. Lastly, we avoided analyzing specimens of M. aequa and *M. lensiformis*, because tests of these species frequently showed significant recrystallization (Figure S2).

## 4 Results

Bulk sediment carbon and oxygen isotopes were determined to refine the stratigraphy and to test whether plankton changes were linked to early Eocene carbon cycle perturbations and warming events.

### 4.1 Bulk Carbon Isotope Records

The δ^13^C values across Site 1051 range between ~0.00% and ~1.50‰, with an average of 0.85‰ (Figure 2, Table S1). From a broad perspective, δ^13^C decreases from the base of the studied interval toward a prominent low at 417.3 mbsf and increases above. Superimposed on this trend, nine negative CIEs, with magnitudes between ~0.20‰ and ~0.70‰, can be identified in our record at Site 1051 labeled as CIE1–CIE9 (Table S1; Figure 2). The bulk sediment δ^13^C record generated by Cramer et al. (2003) partially overlaps the studied interval, from the base up to ~420 mbsf. The two δ^13^C profiles align, although there are slight differences, as can be expected, given reported analytical errors and slightly different sampling depths. The integration of δ^13^C changes with bio‐ and magneto‐stratigraphy provides a powerful means to correlate early Paleogene records from different locations. Most of the CIEs recorded in the studied interval at Site 1051 occurred during Chron C24 (Table S1; Figure 2).

### 4.2 Bulk Oxygen Isotope Records

Bulk carbonate oxygen isotope values at Site 1051 vary between 0.14‰ and −0.95‰ in the early Eocene and between 0.00‰ and −1.11‰ in the early–middle Eocene (Figure 2, Table S1). Two intervals with trends to lower δ^18^O values are found within the main phase of the EECO. The lower interval occurs between 428.78 and 417.30 mbsf and spans CIE5–CIE6b. The most negative values within this interval approach −0.9‰. The upper interval occurs between 408.68 and 390.10 mbsf, which corresponds to the base of the unrecovered interval, and where δ^18^O values reach −0.70‰. Samples above the missing section (377.0–370.4 mbsf) display a mean δ^18^O value of ~0‰, which includes two single‐sample shifts toward −1‰. The mean values of δ^18^O for the early–middle Eocene, therefore, are more positive (~0.5‰) with respect to those of the early Eocene.

Similar to δ^13^C, several negative δ^18^O excursions punctuate the overall δ^18^O trends at Site 1051. Interestingly, these show a clear correspondence to the CIEs (Figure 2). The two most prominent δ^18^O negative shifts are of ~0.8‰ (428.68 mbsf) and ~0.5‰ (406.70 mbsf) and coincide respectively with CIE5 and CIE7b. Furthermore, negative δ^18^O shifts of ~0.3–0.4‰ coincide with most of the other CIEs.

### 4.3 Abundance Changes in *Acarinina*, *Morozovella*, Subbotinids, and Radiolarians

Abundant and diverse planktic foraminifera characterize the studied succession. The population is characteristic of subtropical open‐ocean assemblages and relatively stratified water column due to the occurrence of both surface (morozovellids and acarininids) and thermocline (subbotinids) taxa. Washed residues also include significant numbers of radiolarians throughout, but they markedly increase within the EECO (Luciani et al., 2016; Shipboard Scientific Party, 1998). The meso‐oligotrophic conditions of upper water column may have moved toward more eutrophic conditions during the EECO as the increase of siliceous plankton suggests (see discussion in section 5.5). Planktic foraminifera are recrystallized but are generally free of infilling (Figure S2).

Overall trends in the abundances of planktic foraminiferal genera follow those determined at lower sample resolution (Luciani et al., 2016) but with much greater detail. The most noticeable refinement is that the marked and permanent decline in *Morozovella* abundance precisely coincides with the J event (CIE5). *Morozovella* percentages in samples decrease from a mean value of 43% to 12% across this short interval (Figure 3, Table S2). The loss of *Morozovella* was not compensated by the appearance of the genus *Morozovelloides*, which shared the same ecological preferences with *Morozovella* (Pearson et al., 2006). The *Morozovella* decline was counterbalanced by a prominent increase in *Acarinina* abundance, from a mean value of ~37% below CIE5 to 62% above. The thermocline‐dwelling subbotinids gradually increase their abundance up‐core section, from a mean value of ~15% in the early Eocene to ~25% in the middle Eocene.

Beyond the major switch, several transient variations in the abundance of planktic foraminiferal genera relate to the identified CIEs (Figure 3). This is best realized in the record of *Acarinina* abundance, which shows marked peaks generally corresponding to CIEs. Peaks in the abundance of *Morozovella* are mainly out of phase with those of *Acarinina*.

The mean abundance of radiolarian tests across the studied interval is 27% (Figure 3). However, this abundance increases significantly within the EECO, where it averages ~43.7% and has a peak of 78%. The first prominent increase in radiolarian abundance (65–70%) corresponds to the marked morozovellid decline, and the maximum occurs just before the mostly unrecovered chert interval.

### 4.4 Variations in *Morozovella* Species and Biostratigraphy

The pre‐EECO interval at Site 1051 has a diverse *Morozovella* population with all early Eocene species (e.g., Aze et al., 2011; Pearson et al., 2006) represented (Figure 4). The distribution of *Morozovella* species within samples changes significantly with depth and time (Figure 4). The major switchover in planktic foraminifera is complex at the species level. Preceding CIE‐5, *M. crater* and *M. marginodentata* are the most abundant forms (mean abundance of 9% and 10%, respectively). After this event, *M. crater*, M. aragonensis, and *M. lensiformis* are the most common species (mean abundances of 5%, 3%, and 2%, respectively).

Coincident with CIE5, *M. marginodentata*, *M. subbotinae*, and M. gracilis exhibit large reductions in mean abundance (Figure 4, Table S3). By contrast, *M. lensiformis* remains almost constant, M. formosa is rare (~1%), and M. aragonensis gradually increases upward across the interval investigated. As a consequence of the turnover, *M. crater* and M. aragonensis are the sole species that contribute significantly to the *Morozovella* population in early–middle Eocene sediment at Site 1051 (Figure 4). Transient oscillations in the abundance of different *Morozovella* species are generally in phase, with the exception of *M. marginodentata*.

Moreover, key *Morozovella* events are readily identified and include the following. The top (T) of *M. marginodentata* is recorded at 409.80 mbsf (base C23r) at the upper boundary of Zone E5. The T of M. gracilis and T of M. aequa are recorded at 401.68 mbsf (middle C23r) and 394.10 mbsf (basal C23n), respectively, and within Zone E6/E7a. The base (B) of *Morozovella* caucasica occurs at 397.65 mbsf (middle C23r) and within Zone E6/E7a, although this taxon is quite rare and unevenly distributed above its first appearance. Precise determination of the T of M. formosa and T of *M. lensiforms* is not possible because they apparently fall in the recovery gap between 382 and 390 mbsf.

Most of the *Morozovella* species at Site 1051 became rare to very rare below the level of their definitive disappearances (Figure 4). One can distinguish in some cases, therefore, a top common occurrence (T_c_), which occurs below the aforementioned horizons. As an example, the T_c_ of *M. marginodentata* is found at ~422.4 mbsf and well below its T at 409.8 mbsf. This is important for stratigraphic reasons because rare specimens might be missed. The position of the T_c_ of *M. marginodentata* at Site 1051 is in perfect agreement with the T of this species in the Tethyan Possagno section (Luciani & Giusberti, 2014), whereas the true T of *M. marginodentata* is more consistent with stratigraphic concepts presented by Berggren and Pearson (2005). Due to our detailed counting in samples collected at fairly high temporal resolution, our record of lowest and highest occurrences of *Morozovella* species differs from that reported by the Shipboard Scientific Party (1998).

### 4.5 Variations in Test Size of *Morozovella* Across the Early Eocene

**Figure 5:**
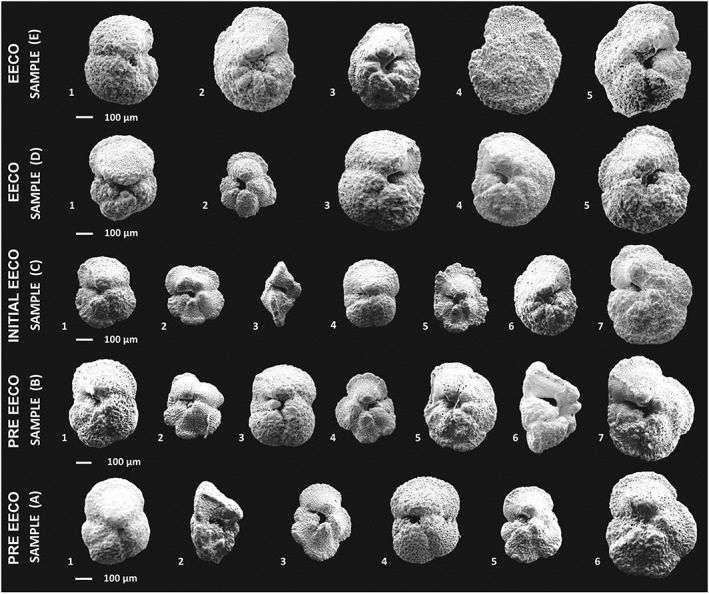
SEM images showing variations in size of representative *Morozovella* species from the >300 μm fraction for the five analyzed “bleaching” samples at Site 1051. Sample A (443.58 mbsf) 1–2: *M. subbotinae*, 3: *M. gracilis*; 4: *M. aequa*; 5: *M. marginodentata*; 6: *M. crater*. Sample B (433.50 mbsf) 1: *M. subbotinae*; 2: *M. gracilis*; 3: *M. aequa*; 4: *M. marginodentata*; 5: *M. aragonensis*; 6–7: *M. crater*. Sample C (425.40 mbsf) 1: *M. subbotinae*; 2–3: *M. gracilis*; 4: *M. aequa*; 5: *M. marginodentata*; 6: *M. aragonensis*; 7: *M. crater*. Sample D (411.80 mbsf) 1: *M. subbotinae*; 2: *M. gracilis*; 3: *M. aequa*; 4: *M. arogonensis*; 5: *M. crater*. Sample E (395.65 mbsf) 1: *M. aequa*; 2–3: *M. aragonensis*; 4–5: *M. crater*. Note that almost the whole *Morozovella* population displays a transitory reduction in size at sample C (initial EECO).

Measurements of the largest *Morozovella* species test size (i.e., the maximum diameter in the ≥300 μm size fraction) exhibit specific trends (Table S4; Figures 4 and 5). A significant reduction in test size occurs in almost all *Morozovella* species in sample C, which comes from just above the J event and the initiation of the EECO. This reduction is particularly marked for *M. lensiformis* (~50 μm), *M. marginodentata* (~30 μm), and *M. subbotinae* (~35 μm) but less evident for M. aequa (~15 μm), M. aragonensis (~20 μm), and *M. crater* (~20 μm). The maximum test size of *M. lensiformis* does not recover above sample C. *Morozovella marginodentata* decreases size from the base upward, passing from 435 μm at sample A to 400 μm at sample C. This species further reduces size, so that it is absent in the ≥300 μm fraction of sample D (Figure 4). By contrast, M. aequa, M. aragonensis, *M. crater*, and *M. subbotinae* almost recover to their pre‐CIE5 test sizes in samples D and E. *Morozovella formosa* records a drop of ~40 μm in test size only at sample E. The test size of M. gracilis shows a significant and permanent drop moving from a mean value of 470 μm to 410 μm starting from the pre‐EECO interval (sample B).

The maximum size of acarininids shows only subtle variations being of 367 μm in sample A, 376 μm in sample B, 373 μm in sample C, 382 in sample D, and 374 μm in sample E.

### 4.6 Stable Isotopes of Different Test Sizes

**Figure 6:**
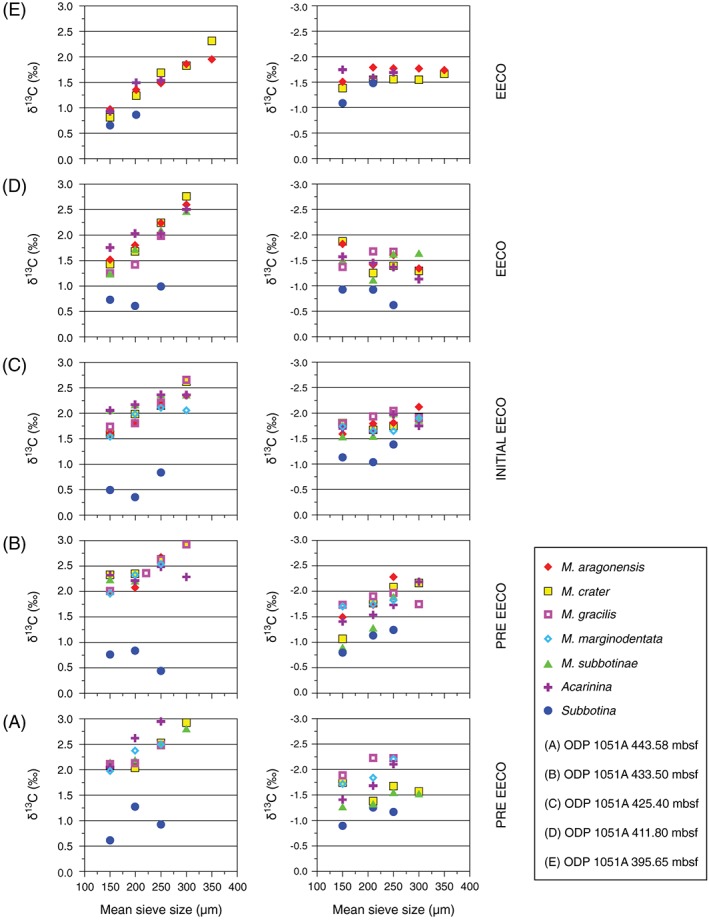
Planktic foraminiferal δ^13^C test size trends from five selected samples (A–E) located below and across the EECO at the ODP Sites 1051. The positions of the analyzed samples are shown in Figures 2 and 3. *Acarinina* includes mixed species with prevalence of *A. esnaensis* and A. interposita in samples A, B, and C and of *A. quetra* in samples D and E, as they are the most abundant and best preserved (not infilled) forms in the respective intervals. *Subbotina* also includes mixed species in which *S. patagonica* and *S. roesnaensis* are mostly represented in all the samples examined. Note the reduction in test size–δ^13^C gradient for *M. marginodentata* and *M. subbotinae* at the initial EECO (sample C). This reduction is coincident with a decrease in maximum test size (see Figures 4 and 5 and discussion in the text) and suggests an episode of bleaching. The δ^18^O compositions of the surface‐dwelling *Morozovella* and *Acarinina* are generally significantly lower (~1‰), except for sample (E), than those of the thermocline‐dwelling subbotinids. However, δ^18^O data at Site 1051 are affected by some recrystallization of plantic foraminiferal test.

As might be predicted from previous work (e.g., D'Hondt et al., 1994; Norris, 1996; Shackleton et al., 1985; Spero & DeNiro, 1987; Spero & Lea, 1993; Wade et al., 2008), the test size relates to the stable isotope composition of certain planktic foraminifera at Site 1051 (Figure 6, Table S1). For most of the examined samples, there is a clear increase in the δ^13^C values of *Morozovella* species with respect to size. This is also true for the samples of mixed *Acarinina* species. However, the δ^13^C–test size gradients of *Subbotina* do not display any clear variation in δ^13^C with respect to different test sizes.

The δ^13^C gradients between small and large tests of *Morozovella* species and *Acarinina* spp. generally exceed 0.7‰. Nonetheless, along with the decrease in their test size, this gradient decreases by ~0.5‰ for samples of *M. marginodentata* and *M. subbotinae* in sample C, slightly above the main reduction in abundance of these species (Figure 6). The variation in the “test size–δ^13^C gradient” recorded by *M. subbotinae* is, however, transient, because this species exhibits a completely restored and even enhanced relationship in sample D. It is not possible to verify whether this occurs for *M. marginodentata*, because this species becomes extremely rare above sample C.

The genus *Acarinina* records as well a significant, though transient, decline in the test size–δ^13^C gradient of samples B and C (Figure 6). Interestingly, M. aragonensis and *M. crater* increased their test size–δ^13^C gradient (~0.5‰) in samples D and E, during the main phase of EECO.

In terms of absolute mean values, subbotinids are characterized by a lower δ^13^C composition (from ~0.5 to ~1.0‰) with respect to acarininids and morozovellids, which have δ^13^C values between ~1.0‰ and ~3.0‰ (Figure 6). A general trend toward lower δ^13^C values is clearly evident within the EECO phase for both *Acarinina* and *Morozovella*, so that values decrease from ~2‰–3‰ in sample A to 0.75‰–2.20‰ in sample E. As a consequence, δ^13^C values of these genera become closer to those of *Subbotina* in sample E.

The δ^18^O values of the size fractions are presented (Figure 6, Table S1) but not rigorously discussed due to some test recrystallization. However, as expected (e.g., Shackleton et al., 1985), the δ^18^O compositions of the surface‐dwelling *Morozovella* and *Acarinina* are generally significantly lower (~1‰), except for sample (E), than those of the thermocline‐dwelling subbotinids.

## 5 Discussion

### 5.1 Early Eocene Carbon Cycle Perturbations at Site 1051

Carbon isotope stratigraphy (Scholle & Arthur, 1980; Shackleton, 1986) provides a powerful means to align stratigraphic sequences across the world at fine‐temporal resolution and allows us to effectively constrain the planktic foraminferal variations recorded at Site 1051.

Well‐resolved bulk sediment δ^13^C records now exist across portions of the EECO at DSDP Site 577 (Shatsky Rise, northwest Pacific; Cramer et al., 2013; Dickens & Backman, 2013; Luciani et al., 2016), ODP Site 1258 (Demerara Rise, western equatorial Atlantic; Kirtland‐Turner et al., 2014), ODP Site 1262 (Walvis Ridge, southeast Atlantic; Zachos et al., 2010), along the Mead and Branch stream sections (New Zealand; Nicolo et al., 2007; Slotnick et al., 2012, 2015), the Possagno quarry outcrop (northeast Italy, Luciani et al., 2016), and the Contessa Road section (central Italy; Coccioni et al., 2012). A detailed benthic foraminifera (*Nuttallides truempyi*) δ^13^C record across the EECO also has been generated at ODP Site 1263 (Walvis Ridge; Lauretano et al., 2016).

When aligned with bio‐ and magneto‐stratigraphic data, the early Eocene bulk sediment δ^13^C record at Site 1051 is remarkably similar to those at other locations, with both the long‐term trends and the superimposed short‐term CIEs (Figures 2 and S1). Following Cramer et al. (2003), CIE1 through CIE5 at Site 1051 represent the H1, H2, I1, I2, and J events. The prominent CIE6b marks the K/X event (e.g., Cramer et al., 2003; Lauretano et al., 2016; Leon‐Rodriguez & Dickens, 2010; Luciani et al., 2016; Slotnick et al., 2012, 2015), also referred as ETM3 (Zachos et al., 2010) or the C24n.1n‐H1 event (Kirtland‐Turner et al., 2014). The minor δ^13^C negative shifts CIE6a and CIE6c immediately below and above this event have been documented at Site 1263 (Lauretano et al., 2016) and in New Zealand sections (Slotnick et al., 2012, 2015). The CIE7a and CIE7b, near the C23r/C24n.1n boundary, can be correlated with the L event(s) initially identified at several sites (Cramer et al., 2003) and subsequently found at other locations (Coccioni et al., 2012; Kirtland‐Turner et al., 2014; Lauretano et al., 2016; Luciani et al., 2016; Slotnick et al., 2012, 2015). The stratigraphic position of CIE8 suggests a correlation with an event named M (Coccioni et al., 2012; Lauretano et al., 2016) and found elsewhere (Kirtland‐Turner et al., 2014; Luciani et al., 2016). Definitely, detailed bulk sediment (and benthic foraminifera) δ^13^C records from widely distributed sections, including now at Site 1051, can be aligned across the EECO, implying a very dynamic global carbon cycle during this time (Kirtland‐Turner et al., 2014; Slotnick et al., 2012). The δ^13^C record at Site 1051 (Figure 2) thus provides a means to correlate with records generated elsewhere.

While not a primary focus of this work, it is worth noting key differences between early Eocene bulk sediment δ^13^C records at Site 1051 and other locations (Figure S1). Mean absolute δ^13^C values at Site 1051 are similar to those at the equatorial Atlantic Site 1258 (Kirtland‐Turner et al., 2014), although the K/X event is not very significant in the latter record. By contrast, the mean bulk sediment δ^13^C values from the Clarence Valley sections (Slotnick et al., 2012, 2015), from Site 1262 (Zachos et al., 2010), and Site 577 (Luciani et al., 2016) exceed those at Site 1051 by nominally 0.4‰, 0.5‰, and 0.6‰, respectively, but the K/X event is prominent. The values from the Tethyan sections of Possagno (Luciani et al., 2016) and Contessa Road (Coccioni et al., 2012) differ still, with bulk sediment δ^13^C records being generally ~0.2‰ lower across the EECO. Overall, bulk sediment δ^13^C records show a clear increase from the North Atlantic and western Tethys (low values), through the South Atlantic and the Indian Ocean, to the Pacific (high values). However, given the paleo‐locations, this signature could also reflect a latitudinal component. The δ^13^C record of bulk sediment at Site 1051 is significantly more positive (~0.8‰) than the benthic foraminiferal *N. truempyi* record at Site 1263 (Lauretano et al., 2016; Figure S1). In any case, the spatial offsets in δ^13^C, both across surface waters and with depth, somewhat resemble those of dissolved inorganic carbon in the modern ocean (Tagliabue & Bopp, 2008).

### 5.2 Bulk Sediment Oxygen Isotopes and TEX_86_: Temperature Changes Across the EECO

The bulk sediment δ^18^O record from Site 1051 traces the general global trend recorded by the benthic foraminferal compilation (Zachos et al., 2001, 2008) and the bulk sediment δ^18^O oxygen profile from DSDP Site 577 (Shatsky Rise; Luciani et al., 2016). All these records show very low values within the EECO. For example, at Site 1051, mean values of δ^18^O for the earliest middle Eocene are significantly more positive (~0.5‰) than those of the early Eocene (Figure 2).

The basic problem with using δ^18^O as a temperature proxy within the studied interval concerns diagenesis. Nannofossil preservation is poor to moderate with specimens marked by overgrowth (Mita, 2001). The planktic foraminifera, though easily recognized, have a “frosty” preservation (Figure S2) that is caused by recrystallization (Sexton et al., 2006). The resulting δ^18^O values of bulk carbonate at Site 1051 not only reflect complications arising from mixed components with different vital effects but also partial recrystallization of calcite that occurred at or beneath the seafloor (e.g., Pearson, 2012; Pearson et al., 2001; Sexton et al., 2006). On the other hand, if carbonate diagenesis occurred within a relatively closed system at the m‐scale (Frank et al., 1999; Matter et al., 1975; Slotnick et al., 2015), the δ^18^O record may still relate to the original composition affected by temperature. This would explain why the bulk carbonate record at Site 1051 resembles those at other locations, including the lows in δ^18^O across the CIEs. At best, however, the bulk sediment δ^18^O record offers qualitative indications for past changes in temperature. These changes clearly indicate warmer temperatures within the EECO.

Our TEX_86_‐derived temperature for the J event is of ~32.6°C to ~36.0°C (see supporting information) at ~53 Ma and is comparable to that determined for the PETM in North Atlantic shallow‐marine sections at Bass River (33.5°C; Sluijs et al., 2007) and at Wilson Lake (35.7°C; Zachos et al., 2006). As suggested by benthic foraminiferal δ^18^O records (Lauretano et al., 2016; Zachos et al., 2008), global temperatures during the short‐term PETM event and the longer term EECO may have been comparable. The terrestrial influx is minimal at Site 1051 since the location is relatively far from land, a concept supported by the very low BIT value (0.126; see supporting information). It must be highlighted, however, that our TEX_86_ measurement is influenced by the scarce preservation of GDGT, and that a proper calibration for TEX_86_‐derived SSTs, especially during the Eocene, is a source of current debate (Ho & Laepple, 2016; Ingalls, 2016; Kim et al., 2010). However, records of δ^18^O‐derived sea surface‐water temperatures from the tropical Tanzanian site at 53.2 Ma as high as 30.6°C to 33.3°C were obtained by exceptionally well‐preserved (“glassy”) mixed‐layer planktic foraminifera (Pearson et al., 2007). These results appear comparable with our TEX_86_‐derived temperature (Anagnostou et al., 2016). In conclusion, due to the characteristics of the sediments analyzed, we cannot reconstruct for Site 1051 reliable values of paleotemperature across the EECO onset when the main drop in morozovellid abundance occurred.

### 5.3 Variability in Early Eocene Planktic Foraminiferal Populations

Early Eocene planktic foraminifera assemblages at Site 1051 exhibit both permanent and transitory changes, the latter largely coincident with major carbon and oxygen isotope excursions (Figures 2 and 3). However, it is first worth considering carbonate dissolution, as this can modify the composition of planktic foraminiferal assemblages significantly (e.g., Bé et al., 1975; Berger, 1970; Nguyen et al., 2009; 2011; Petrizzo et al., 2008; Thunell & Honjo, 1981). Events with stable isotope anomalies in the early Eocene generally were marked by low carbonate contents in deep‐sea settings (e.g., Leon‐Rodriguez & Dickens, 2010; Stap et al., 2009; Zachos et al., 2005), presumably because massive and rapid carbon input to the ocean and atmosphere shoaled carbonate saturation horizons (Dickens et al. 1997; Kump et al., 2009; Zeebe et al., 2009). However, the *F* index dissolution proxy (Figure 3) exhibits low values (<10%) across most of the studied section, especially within the EECO, although moderate increases are found at the I‐1 (~30%) and J (~40%) events. This suggests that changes in the foraminiferal assemblages represent genuine biotic trends rather than changes in dissolution.

Our high‐resolution record clearly demonstrates that the long‐term permanent *Morozovella* decline in abundances precisely corresponds to the J event. The morozovellid drop occurred within an estimated time interval of 2–4 kyr, between the sample coinciding with the J event and the sample immediately above. Acarininid increase started slightly below (~5 kyr).

The short‐term peaks in *Acarinina* abundance at Site 1051 are similar in timing and absolute values to those observed in the Tethyan Possagno section, where they also mainly correspond to negative CIEs (Luciani et al., 2016). Studies of Paleogene sediment suggest that the genus *Acarinina* was more resistant to dissolution than *Morozovella* and that both these were more resistant than subbotinids (Nguyen et al., 2009, 2011; Petrizzo et al., 2008). Carbonate dissolution clearly occurred during some of the CIEs, especially for the Possagno section where *F* index reaches values as high as 60–70% within the EECO. Such dissolution may have partly amplified the foraminiferal assemblage changes (Luciani et al., 2016). However, at the Tethyan Terche section, D'Onofrio et al. (2014, 2016) recorded pronounced spikes in *Acarinina* abundance at the ETM2, H1, and I2 events where planktic foraminferal assemblages are less biased by dissolution. We therefore consider that the increased abundances of *Acarinina* during the CIEs, and certainly across the major switch following the J event, reflect genuine responses of low‐latitude planktic foraminferal populations to early Eocene variations in surface water properties.

### 5.4 Paleoecology of Early Eocene Planktic Foraminifera at Site 1051

The distinct increase in δ^13^C with test size for all analyzed *Morozovella* species and *Acarinina* spp. (Figure 6) confirms that both these genera had photosymbionts (e.g., D'Hondt et al., 1994; Norris, 1996; Shackleton et al., 1985). By contrast, the absence of test size–δ^13^C gradients in *Subbotina* specimens is consistent with asymbiotic ecology (e.g., Norris, 1996; Pearson et al., 1993). The differences in the mean δ^13^C values for *Morozovella* and *Acarinina* with respect to *Subbotina* give evidence for different depth habitats. The relatively high δ^13^C values for the former suggest a mixed‐layer habitat, while relatively low δ^13^C values for the latter suggest a thermocline habitat (e.g., Boersma et al., 1987; Pearson et al., 2006; Shackleton et al., 1985, and references therein). While the above is not surprising given previous work (also Berggren & Norris, 1997; Lu & Keller, 1996), we provide for the first time stable isotope data for early Eocene *M. subbotinae*, *M. causasica*, *M. crater*, and M. formosa.

Our stable isotope data provide new insights on the paleobiology of morozovellid species and on their relationships with *Acarinina* in the early Eocene mixed‐layer habitat. In particular, *Morozovella gracilis* generally shows lower δ^18^O values with respect to other morozovellid species (Table S1, Figure 6). This suggests slightly different temperatures and that it possibly occupied shallower depth habitat or warmer season. Pearson et al. (2006, pp. 369–370) stated that the morphological differences between *M. marginodentata* and M. gracilis are sometimes subtle since “intergradation of the typical morphologies” of the two species can be found “in most early Eocene (sub)tropical fossil assemblages.” Blow (1979) even suggested that *M. marginodentata* could have been an “extreme phenotype” of M. gracilis. The δ^18^O differences might reinforce the hypothesis that *M. marginodentata* and M. gracilis were two separate morphospecies. Certainly, in modern planktic foraminiferal fauna, slightly different morphotypes often mask considerable genetic diversity (e.g., Darling et al., 2006; Darling & Wade, 2008).

In most of the studied samples, *Acarinina* spp. exhibit slightly greater δ^13^C values with respect to most *Morozovella* species (Figure 6). The δ^13^C feature has been found in examinations of other late Paleocene and early Eocene foramininiferal assemblages (e.g., Boersma et al., 1987; Quillévéré et al., 2001; Shackleton et al., 1985). *Acarinina* may therefore have lived even slightly shallower than *Morozovella* in the mixed‐layer habitat. However, δ^18^O values are only partly consistent with δ^13^C values, perhaps due to test recrystallization that occurred at or beneath the seafloor. This can explain the δ^18^O values exhibited by the surface‐water dwellers *Morozovella* and *Acarinina* that are close to those of the thermocline dweller *Subbotina*. Given the differences in δ^13^C of *Morozovella* and *Acarinina* and the out‐of‐phase fluctuations in the abundances of these two genera at Site 1051 and other locations (e.g., D'Onofrio et al., 2016; Luciani et al., 2016), one might suggest slight differences in their ecological behavior that could induce competition within the overall mixed‐layer habitat. Interestingly, indications of interspecies competition within the photic zone has been documented by Birch et al. (2012) for the late Paleocene symbiont bearing planktic foraminifera from Walvis Ridge (Southern Atlantic).

The ~0.5‰ reduction in the δ^13^C gradient between mixed‐layer dwellers (*Acarinina* and *Morozovella*) and thermocline dwellers (subbotinids) over the EECO (Figure 6) warrants discussion. This has been documented in previous studies of planktic foraminifera (e.g., Boersma et al., 1987; Bralower et al., 1995; Lu & Keller, 1996), although with generally poorer stratigraphic framework. Moreover, the decline in the δ^13^C gradient manifests between shallow‐water dwellers and benthic foraminiferal records (e.g., Boersma et al., 1987; Bralower et al., 1995; Lu & Keller, 1996), which generally track the subbotinid records. One possible mechanism for this is an increase in the vertical mixing of the oceans so that less respired carbon dioxide accumulated at depth (e.g., Hilting et al., 2008). The primary problem with this idea is that records of bulk carbonate, which principally consist of photosynthetic calcareous nannofossils (e.g., Regellin et al., 2015; Stap et al., 2009; Stoll, 2005), track those of benthic foraminifera and subbotinids. This suggests that the reduction in the δ^13^C gradient reflects a change in *Acarinina* and *Morozovella* lifestyle, and how they record carbon isotopes, rather than a change the dissolved inorganic carbon of shallow and deep‐water masses. One possibility is that with sustained warmth or other unfavorable changes in surface waters during the EECO, *Acarinina* and *Morozovella* slowly reduced their photosymbiont relationship, perhaps by living in slightly deeper water depths, where photosynthetic activity is lower. The reduction in the maximum test sizes of M. formosa, M. gracilis, *M. lensiformis*, and *M. marginodentata* may support this hypothesis because attainment of large test sizes in symbiotic species is an indication of ecological success as a consequence of symbiont nutrition and such size reduction could imply symbiosis became less important for sustenance.

### 5.5 Bleaching at the Onset of the EECO

A primary purpose of the present study was to address the issue of bleaching as a principal mechanism for the relatively rapid and permanent decline in the photosymbiont‐bearing *Morozovella* that occurred at the onset of the EECO (Luciani et al., 2016). We are aware that possible stressors inducing loss of photosymbiosis are associated with the EECO, including extreme warmth, higher pCO_2_ (e.g., Bijl et al., 2009; Hollis et al., 2012; Huber & Caballero, 2011; Inglis et al., 2015; Pross et al., 2012; Zachos et al., 2008), and lower surface‐water pH (Anagnostou et al., 2016).

Our data records a decrease in the δ^13^C–test size gradient for morozovellids just after the J event suggestive of bleaching. Interestingly, a reduced δ^13^C–test size gradient occurs within *Acarinina* spp. at the same interval despite their apparent increase in abundance (Figure 6). One possibility for this reduction in gradients was a change in the type of hosted algal symbionts. Living planktic foraminifera bearing chrysophyte symbionts have a δ^13^C–test size gradient much lower than those hosting dinoflagellates (e.g., Bornemann & Norris, 2007; Hemleben et al., 1989). Some laboratory experiments on modern foraminifera indicate that they can change their algal symbiont preferences, but this observation so far only pertains to genetic subgroups of dinoflagellates (e.g., Shaked & de Vargas, 2006). There is no indication that planktic foraminifera can change their symbiont type from dinoflagellate to chrysophyte symbionts during their life cycle or between succeeding generations (e.g., Gast & Caron, 1996; Hemleben et al., 1989). Importantly, at Site 1051 and at the initiation of EECO, there is also a significant decrease in maximum test size of *Morozovella* (Figures 4 and 5). To account for the observations, the simplest hypothesis is that morozovellids reduced their photosymbiont relationship at the onset of the EECO. The algal symbionts may have been lost due to morozovellid migration to deeper waters, where light radiation for algal photosynthesis becomes attenuated. Inhabiting slightly deeper waters would have allowed morozovellids to maintain surrounding temperatures when the mixed layer became exceptionally warm. Alternatively, changes in nutrient regimes may be involved. There is a significant radiolarian increase within the main phase of the EECO at Site 1051, coincident with the morozovellid collapse. The abundant siliceous microfossil assemblages from ODP Site 1051 indicates an overall increase in the trophic state of surface waters. Siliceous components include also diatoms (Shipboard Scientific Party, 1998) that further reinforce the hypothesis of enhanced eutrophication. High nutrient availability also may have caused stress for the oligotrophic morozovellids. Surface‐water eutrophy during the EECO at the studied site seems also supported by the early Eocene calcareous nannofossil assemblages that were dominated by Coccolithus pelagicus (Mita, 2001), a taxon with affinities to warm and eutrophic surface waters (e.g., Agnini et al., 2006, 2007, 2009; Dedert et al., 2012; Fornaciari et al., 2007; Newsam et al., 2017; Perch‐Nielsen, 1981; Tremolada & Bralower, 2004). Interestingly, high‐productivity intervals characterized by increased in siliceous plankton were recorded at Site 1051 (Moebius et al., 2015; Witkowski et al., 2014) at the peak of Middle Eocene Climatic Optimum, a longer‐lived warming event of ~500 kyr that occurred at ~40 Ma (e.g., Bohaty & Zachos, 2003; Bohaty et al., 2009). The eutrophication in the slope of the adjacent North American continental margin has been considered as triggered by intensified hydrological cycle during the climatic warmth of the Middle Eocene Climatic Optimum, leading to an increase in riverine input into the ocean (Moebius et al., 2015; Witkowski et al., 2014). We can speculate that also the intense warming at the EECO may have lead to similar conditions at Site 1051. Nevertheless, upwelling of nutrient‐rich waters also commonly gives rise to high surface productivity. Although the western margin of the Atlantic Ocean is not an upwelling locality at present, the Blake Plateau may have been the locus of upwelling during the late–middle Eocene (Wade et al., 2001). Thus, shifts in the location of upwelling or an increase in upwelling intensity due to change of direction in the coastal wind system could have induced to transient modification in local productivity at this site. Upwelling conditions are typically associated with extreme eutrophy that usually forces marked change in planktic foraminiferal assemblages and favored flourishing of opportunist taxa. As we do not observe such striking changes in the assemblages at Site 1051, the upwelling hypothesis seems unlikely.

Considering that acarininids reduced their δ^13^C–test size gradient but not abundance and size, it is possible that this group may have been able to better adapt than morozovellids to more eutrophic waters where symbiosis is not necessary. There are several lines of evidence to suggest that acarininids were better suited to higher trophic conditions; for example, they evolved at the more eutrophic waters of high latitudes (e.g., Quillévéré et al., 2001). The records from the early Eocene Tethyan sections also indicate their tolerance to relatively eutrophic waters (e.g., Agnini et al., 2009; D'Onofrio et al., 2016; Luciani et al., 2007).

In summary, we record here a likely bleaching episode at the initiation of EECO from a subtropical location. This bleaching event occurred at the time of the permanent low‐latitude morozovellid collapse in abundance, but it affected also the acarininids that proliferated afterward (Luciani et al., 2016). Moreover, the postulated bleaching episode was transitory, because photosymbiotic activity in *Morozovella* and *Acarinina* appears to have recovered within the main phase of the EECO. We cannot therefore assign reduced photosymbiotic activity as the sole cause for morozovellid decline. However, surface water changes at the start of the EECO disturbed ecosystem dynamics sufficiently to have enabled acarininids to eventually outcompete morozovellids.

### 5.6 Morozovellid Species Response at the Onset of the EECO

The permanent switch in photosymbiont bearing morozovellids at the onset of EECO was complex, at least at Site 1051 (Figures 4 and 5). Notably, the species M. gracilis, *M. lensiformis*, *M. marginodentata*, and *M. subbotinae* were dominant before the J event, and the large reductions in these four species give rise to the major morozovellid collapse. For M. gracilis and *M. marginodentata*, the J event also marks a decrease in their δ^13^C–test size gradient and a long‐term shrinkage in their maximum diameter, even after pre‐event photosymbiotic relationships were apparently restored. For M. formosa and *M. lensiformis*, while the maximum size drops, we cannot establish whether they were affected by a transient or permanent decrease in photosymbiotic activity, because we were not able to obtain reliable stable isotope data. A decrease in abundance and test size has been documented in members of the genus *Morozovelloides* just before its evolutionary disappearance in the late–middle Eocene (Wade & Olsson, 2009). In this case, the reduced test size was attributed to a permanent loss in algal photosymbiosis.

Organisms are equipped with a certain adaptive plasticity to face environmental challenges, and thus, different phenotypes may develop in response to imposed conditions (e.g., Mayr, 1970; Schmidt et al., 2006). The species M. formosa, M. gracilis, *M. lensiformis*, and *M. marginodentata* may have reacted with morphological changes through ecophenotypic plasticity within their adaptive range, resulting in the observed test size reduction. For example, as a strategy to face increased surface water temperature, average individuals within foraminiferal populations could have moved to slightly deeper water depths, but where light levels were lower and photosymbiont activity would decrease. Even though we have evidence that M. gracilis, *M. marginodentata*, and *Acarinina* spp. restored the algal photosymbiosis after the J event, the general reduction in δ^13^C gradient between the mixed‐layer dwellers and *Subbotina* spp. through the EECO suggests that these genera may have indeed moved to slightly deeper depth or become less reliant on photosymbionts permanently.

However, potential causes explaining the reduced size across the J event and within the EECO are manifold, perhaps interrelated, and they may include, beside reduced symbiotic relationship, changes in primary production, salinity, temperature, and decrease in oxygen levels (e.g., Hallam, 1965; Schmidt et al., 2006). Protists require more life resources, such as oxygen and nutrients, when temperature increases, because their metabolism accelerates (e.g., O'Connor et al., 2009), but in warm waters the concentrations of dissolved oxygen decreases. Thus, a strategy to optimize resource uptake is to enlarge surface area/volume ratio by reducing the cell mass and therefore the test size (e.g., Atkinson et al., 2003). Changes in ocean chemistry also may have affected morozovellid calcification and explain to some extent the observed test size reduction. Recent culturing and open ocean observations suggest that acidification can affect variably sized foraminifera differently, such that larger planktic foraminifera preferentially reduce their calcification (Henehan et al., 2017). We cannot exclude that morozovellids may have decreased their maximum size as a consequence of a drop in pH, because there are several short‐term negative CIEs within the EECO probably signifying addition of CO_2_ to the ocean and atmosphere (e.g., Dickens et al., 1997; Zeebe et al., 2009).

Our record shows that only a limited number of *Morozovella* species at Site 1051 decreased their test size at the start and through the EECO suggesting dissimilar levels of tolerance to environmental and climatic changes. In particular, M. aragonensis and *M crater* appear largely unaffected by the J event, becoming the dominant *Morozovella* species afterward. Here it is interesting to realize that these two species likely evolved from *M. lensiformis* (Aze et al., 2011; Blow, 1979; Pearson et al., 2006), and they may have succeeded because they could tolerate the changes whereas *M. lensiformis* became extinct.

Acarininids do not appear affected by reduction in test size across the EECO, although our data are restricted to the genus level. This demonstrates, together with their marked increase in abundance, the high tolerance and adaptability of acarininids to the changed environmental conditions.

## 6 Summary and Conclusions

To understand the striking *Morozovella* abundance decline near the onset of the EECO at low‐latitude sites (Frontalini et al., 2016; Luciani et al., 2016), we have refined the stratigraphy at ODP Site 1051 and documented changes in planktic foraminiferal assemblages and their geochemistry. The work began with an idea of possible cause: the loss of photosymbionts; the work concludes with the following main results:

1. As in other early Eocene sections, the bulk carbonate δ^13^C curve at Site 1051 provides a detailed stratigraphic means to relate global carbon cycle changes, including CIEs, to paleoceanographic changes, notably in this case planktic foraminiferal turnovers. Such correlation proves that the marked *Morozovella* decline precisely coincided with the J event, at least at Site 1051. The species M. gracilis, *M. lensiformis*, *M. marginodentata*, and *M. subbotinae* were dominant before the J event, and large reductions in the abundance of these four species, which ultimately disappeared within the EECO, give rise to the major morozovellid collapse. The drop in their abundance was not counterbalanced by an increase in *Morozovella* species that survived into the middle Eocene.
2. Short‐term fluctuations in foraminiferal abundance at Site 1051 closely relate to shifts in bulk carbonate δ^13^C. In general, peaks in *Acarinina* abundance and lows in *Morozovella* abundance coincide to early Eocene CIEs and the EECO, similar to what has been observed in several Tethyan successions (e.g., Agnini et al., 2009; D'Onofrio et al., 2016; Frontalini et al., 2016; Luciani et al., 2007, 2016). We consider that fluctuations in the abundances of *Acarinina* and *Morozovella* during CIEs reflect genuine responses of low‐latitude planktic foraminferal populations to early Eocene variations in surface water, although dissolution may enhance the signal. This finding, together with the minor differences in their stable isotope composition, suggests some dissimilarity in the ecological behavior of the two genera within their shared mixed‐layer habitat.
3. The algal–symbiont relationship for morozovellids seems to have decreased following the J event, as indicated by a reduction in the δ^13^C–test size gradient. However, this “bleaching” was transitory and also affected the acarininids, which proliferated after the J event. The sudden switch in planktic foraminiferal genera during the early Eocene was a significant evolutionary change in the marine biota, but loss of photosymbionts cannot have been the primary causal mechanism. Seemingly, *Acarinina* were better adapted than most species of *Morozovella* for the competition of life resources during the EECO. Notably, the bleaching episode is not related to any extinction of species.
4. The δ^13^C gradient between mixed‐layer dwellers (*Acarinina* and *Morozovella*) and thermocline dwellers (subbotinids) shows a ~0.5 ‰ reduction over the EECO. A possible explanation for this reduction is that it reflects a change in *Acarinina* and *Morozovella* lifestyle, such that, while still carrying photosymbionts, they moved to slightly deeper waters. This interpretation would explain the similarity of trends in δ^13^C records of benthic foraminifera, subbotinids, and bulk carbonate, the latter which largely consists of photosynthetic calcareous nannofossils. With such a view, *Acarinina* and *Morozovella* reduced their reliance on photosymbionts or moved to slightly deeper water depths, where photosynthetic activity is lessened, perhaps due to sustained warmth or other changes in surface waters during the EECO.
5. The reduction in morozovellid test size is not limited to the bleaching episode as demonstrated by analyses of *M. lensiformis*, M. gracilis, and *M. marginodentata*. These species did not recover their maximum test size, even after having restored the photosymbiotic relationship. In addition to bleaching, the decrease in planktic foraminiferal test size might relate to the crossing of an ecological threshold. For example, a reduction in the cell mass and test size could have been a mechanism to optimize resource uptake in extremely warm surface waters. However, not all *Morozovella* species at Site 1051 contracted their test size at the start and through the EECO. This suggests that various *Morozovella* species had different tolerances to environmental and climatic changes.

## Supporting information



Supporting Information S1

Figure S1

Figure S2

Table S1

Table S2

Table S3

Table S4

Table S5

## Appendix A Taxonomic List of Planktic Foraminiferal Species Cited in Text and Figures

### A.1

*Acarinina cuneicamerata* (Blow, 1979).

*Acarinina esnaensis* (LeRoy, 1953).

*Acarinina interposita* (Subbotina, 1953).

*Acarinina quetra* (Bolli, 1957).

*Astrorotalia palmerae* (Cushman and Bermúdez, 1937).

*Morozovella aequa* (Cushman and Renz, 1942).

*Morozovella aragonensis* (Nuttall, 1930).

*Morozovella caucasica* (Glaessner, 1937).

*Morozovella crater* (Hornibrook, 1958).

*Morozovella formosa* (Bolli, 1957*)*.

*Morozovella gracilis* (Bolli, 1957).

*Morozovella lensiformis* (Subbotina, 1953),

*Morozovella marginodentata* (Subbotina, 1953).

*Morozovella subbotinae* (Morozova, 1939).

*Subbotina roesnaensis* (Olsson and Berggren, 2006).

*Subbotina patagonica* (Todd and Kniker, 1952).
