# Supplementary material for: Did Photosymbiont Bleaching Lead to the Demise of Planktic Foraminifer Morozovella at the Early Eocene Climatic Optimum?
Source: Paleoceanography. 2017 Nov 6;32(11):1115–36. doi: 10.1002/2017PA003138 (PMC5784393; doi:10.1002/2017PA003138)
Supplement: Supplementary file 1 — Supporting Information S1 [file PALO-32-1115-s003.docx]

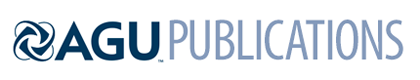


*Paleoceanography*

Supporting Information for

**Testing photosymbiont bleaching in planktic foraminifera**

**at the start of the Early Eocene Climatic Optimum**

Valeria Luciani^1^, Roberta D’Onofrio^1^, Gerald Roy Dickens^2^, Bridget Wade^3^

^1^ Department of Physics and Earth Sciences, Ferrara University, via G. Saragat 1, 44121, Italy: valeria.luciani@unife.it, dnfrrt@unife.it

^2^ Department of Earth Science, Rice University, Houston, TX 77005, USA: jerry@rice.edu

^3^ Department of Earth Sciences, University College London, Gower Street, London, United Kingdom: b.wade@ucl.ac.uk

**Contents of this file**

Text S1. TEX_86_ Methods and Results

Figures S1 to S3

**Additional Supporting Information (Files uploaded separately)**

Dataset Tables S1 to S4

**Table S1.** Carbon and oxygen stable-isotope data from bulk carbonate, restricted size-fraction monospecific specimens of *Morozovella* and from restricted size-fraction specimens of *Acarinina* spp. and *Subbotina* spp. across the early Eocene at the ODP Site 1051A.

**Table S2.** Relative abundances (%) of planktic foraminiferal genera, radiolarians and fragmentation index (*F index*, %) at Site 1051A. In Italic data from Lucani et al. [2016].

**Table S3.** Relative abundances (%) of *Morozovella* species at Site 1051A.

**Table S4.** Test-size diameters of *Morozovella* species and *Acarinina* genus from the >300 µm fraction at Site 1051A.

**Introduction**

This supporting information includes specific description on the adopted TEX_86_ methods and results and provides two additional figures briefly discussed in the main article.

**Text S1. Tetraether Lipid and BIT Index Analysis for the early Eocene at Site 1051**

**Methods**

The TEX86 proxy for paleotemperature reconstruction is based on the evidence that marine *Thaumarcheota* produce isoprenoid glycerol dialkyl glycerol tetraethers (GDGTs) and the distribution of these compounds changes with temperature [e.g. Uda et al., 2001; Boyd et al., 2011]. Considering that marine *Thaumarcheota* live primarily at water depths of 0-200 meters, the TEX86 index has been developed to reconstruct SSTs based on the relative abundance of isoprenoid GDGTs [e.g., Schouten et al., 2002].

Ten bulk sediment samples of ~20 cm3 each were selected from the studied section and examined for their TEX86 values at the Utrecht University GeoLab. Due to the general scarcity of lipids in these samples, an additional “multi-sample” (~60 g r) made by combining four samples spaced 4 cm-apart –within the J event was analyzed. To extract the isoprenoid GDGTs and evaluate their abundances we follow the methods for samples preparations shown in Schouten et al. (2002, 2013) and De Jonge et al. (2014). Prior to lipid extraction, samples were freeze-dried for 48 hours and powdered using mortar and pestle. Lipids from the ten were extracted from 8-10 grams of sediment with dichloromethane (DCM):methanol (MeOH) (9:1, v/v) using a Dionex accelerated solvent extractor (ASE 200) at a temperature of 100°C and under a pressure of 7.6-106 Pa. A larger amount of sediments (~60 g) was instead analysed for the multi-sample. In this case compounds were extracted with preliminary samples a soxhlet extractor using the same solvent mixture (DCM:MeOH, 9:1, v/v). The lipid extracts from both the tests were dried under a N2 stream then separated into an apolar, ketone and polar fraction by column chromatography using an activated Al2O3 column. Eluting was done with hexane:DCM (9:1, v/v), hexane:DCM (1:1, v/v) and DCM:MeOH (1:1, v/v), respectively. Ninety-nine nanograms of a synthetic C46 GDGT-standard were added to the polar fraction, containing the GDGTs. After drying under N_2_ stream, the polar fraction was dissolved in hexane:isopropanol (99:1, v/v) to a concentration of ~3 mg/mL and filtered through a 0.45 μm PTFE (polytetrafluoroethylene) filter. Quantification of GDGTs from the polar fractions was conducted with an HPLC/APCI-MS (high performance liquid chromatography/atmospheric pressure chemical ionization-mass spectrometry) using an Agilent 1100 series LC/MSD SL (liquid chromatography/mass spectrometry) and following methods described by De Jonge et al., (2014).

TEX_86_ values were obtained from the abundance of isoprenoid GDGTs according to *Schouten et al.* [2002]:

TEX_86_= [GDGT2+GDGT3+ Crenarchaeol]**/**[GDGT1+GDGT2+GDGT3+ Crenarchaeol]

where GDGTs 1**–**3 indicate compounds containing 1**–**3 cyclopentyl moieties, and cren′ refer to the regioisomer of crenarchaeol.

The BIT (branched isoprenoid tetraether) index is considered a proxy for the relative amounts of terrestrial and marine tetraethers in organic matter, as the ratio can bias TEX_86_ SST estimates [*Schouten et al*., 2013]. We calculated the BIT index for the multi-sample at the J event CIE-peak as follows [*Hopmans et al*., 2004]:

BIT=[GDGT I]+[GDGT II]+[GDGT III]/[GDGT I]+[GDGT II]+[GDGT III]+[Crenarchaeol]

**Results: TEX_86_ Record and BIT Index**

Isoprenoidal GDGTs containing cyclopentane rings are very scarce throughout the studied interval. All 10 individual samples analysed have concentrations below the detection limit [*Schouten et al.,* 2002; 2013]. The sole valuable datum comes from the combined multi-sample at the peak of CIE-5 (the J event). Despite analyzing a large amount of sediment (~60 g) for this sample, concentrations of some GDGTs, such as the GDGT-1 and GDGT-3, remain only close to the detection limit. On other hand, crenarcheol and its regioisomers, GDGT-4 and GDGT-4´, are particularly abundant. The TEX_86_^H^ value determined for this sample is 0.915, and the BIT-value obtained for this combined sample is very low (0.126).

There is an ongoing debate on which calibration should be used to derive temperatures from TEX_86_  values during warm climates of early Paleogene [*Liu et al.,* 2009; *Keating-Bitonti et al*., 2011; *Hollis et al*., 2012; *Bijl et al.,* 2013; *Schouten et al*., 2013]. We decided therefore to convert our TEX_86_ datum from the J event at Site 1051 using both the logarithmic equation presented by *Kim et al.*, [2010]:

Equation1: SST=68.4×log (TEX_86_)+38.6

and the non-linear relationship presented by *Liu et al*. [2009]:

Equation2: SST=50**.**475─16**.**332(1**/**TEX_86_).

Sea surface temperatures during the J event would thus be ~36.0°C according to the first calibration and 32.6°C according to the second equation. For comparison, modern long-term average monthly SSTs in the region range between 21 and 28°C (<https://iridl.ldeo.columbia.edu/SOURCES/.LEVITUS94/>).

The BIT value obtained is 0.126.

Figure S1. Correlation of carbon isotope records spanning the early Eocene from several Sites worldwide distributed. These are: the Branch Stream (New Zealand) [Slotnick et al., 2015], the Possagno (north-eastern Italy) [Luciani et al., 2016] and Contessa Road section (central Italy) [Coccioni et al., 2012], the ODP Sites 1051 (Blake Nose, this study), 1258 (Demerara Rise) [Kirtland-Turner et al., 2014], 1263 (Walvis Ridge) [Lauretano et al., 2016], 1262 (Walvis Ridge) [Zachos et al., 2010] and the DSDP Site 577 (Shatsky Rise) [Cramer et al., 2003; Luciani et al., 2016]. Where possible, magnetostratigraphy, lithology and planktic foraminiferal zonation are given. Records from Cramer et al. [2003] at Sites 577 and 1051 are in black colour. At Site 577 δ^13^C data are aligned following Dickens and Backman [2013]; orange colour refers to hole 577A and red colour to hole 577A. Carbon isotope excursions (CIEs) are highlighted with pink bands and labelled following the alphabetical order of Cramer et al. [2003]. Original labels used by different authors are shown in brackets. Dashed bands indicate events not consistently correlated. Correlation problems are mainly related to different resolution of δ^13^C records and sedimentation rates. A major shift in frequency and amplitude of CIEs appears to have happened during the EECO.


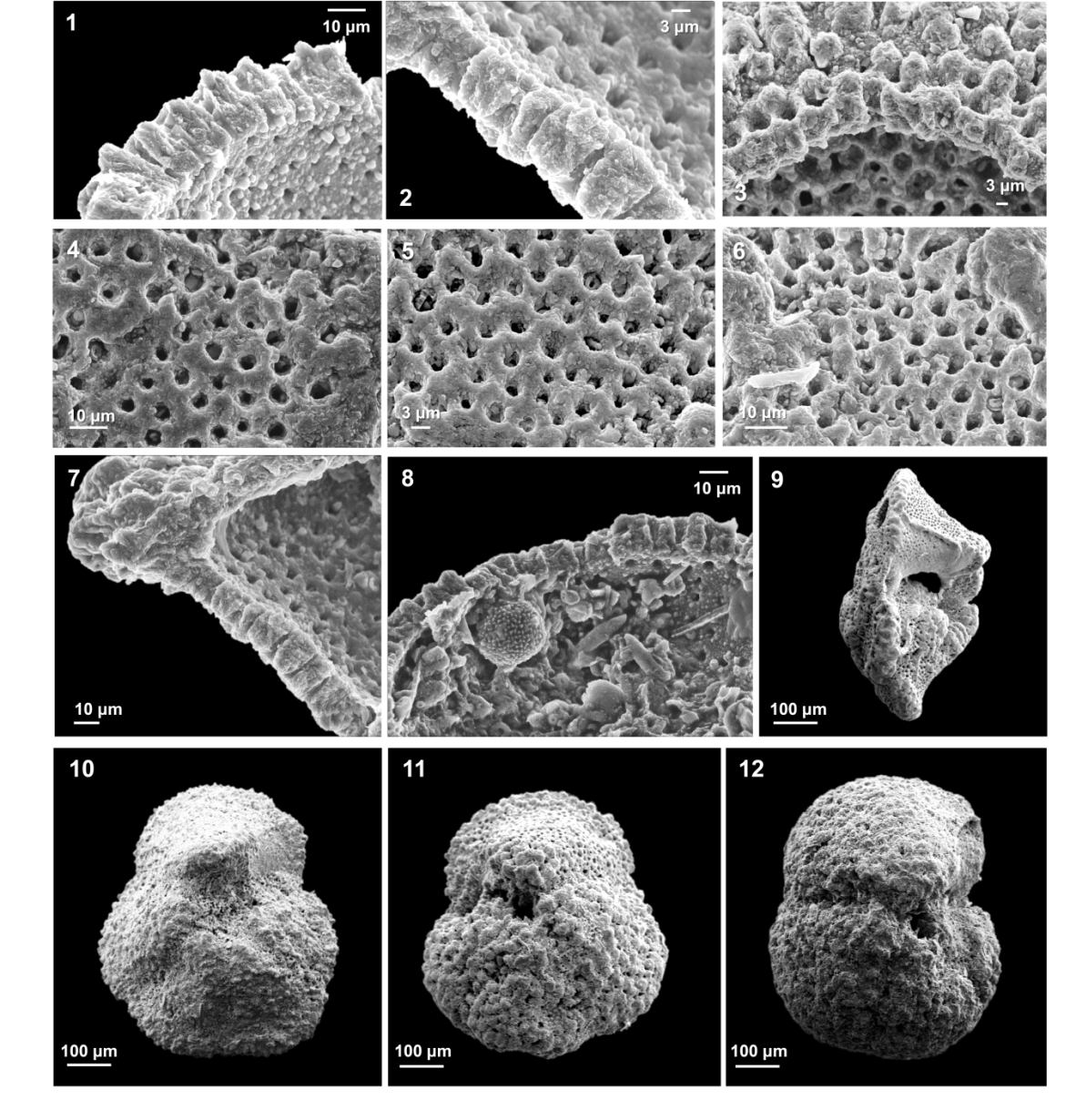


**Figure S2.** Preservation of early Eocene planktic foraminifera (Zones E4−E6/7a) at the ODP Site 1051A. 1−6: SEM images of wall textures showing different degrees of “frosty” preservation (as referred to their appearance at stereomicroscope according to Sexton et al., [2006]). Broken tests from: (1) *Acarinina soldadoensis* (sample 437 mbsf), (2) *Morozovella formosa* (sample 422.9 mbsf), (3) *Subbotina patagonica* (sample 437 mbsf). Images show micron-scale diagenetic alteration even obscuring the relief of the vertical pore pits in some cases. (4) Wall-textures of *A. soldadoensis* (sample 437 mbsf), (5) *M. formosa* (sample 410.8 mbsf), (6) *S. patagonica* (sample 405.22 mbsf) showing variable amounts of secondary calcite. 7−10: examples of infilled and unfilled specimens. (7) Broken test of unfilled *M. formosa* (sample 422.9 mbsf) (8) broken test of *A. soldadoensis* (sample 437 mbsf) infilled with large crystals of secondary calcite; (9) entire specimen of *M. formosa* (sample 410.8 mbsf) with a completely clean aperture denoting that test is potentially unfilled; (10) entire specimens of *M. crater* (sample 405.22 mbsf) with sealed aperture denoting that test is completely infilled. 11−12: Examples of significantly recrystallized test of *M. lensiformis* (11, sample 395.10) and *M. aequa* (12, sample 395.10 mbsf) both displaying replacement of surface pustules by coalescent large crystals.
