# Supplementary figures and images for: Did Photosymbiont Bleaching Lead to the Demise of Planktic Foraminifer Morozovella at the Early Eocene Climatic Optimum?
Source: Paleoceanography. 2017 Nov 6;32(11):1115–36. doi: 10.1002/2017PA003138 (PMC5784393; doi:10.1002/2017PA003138)

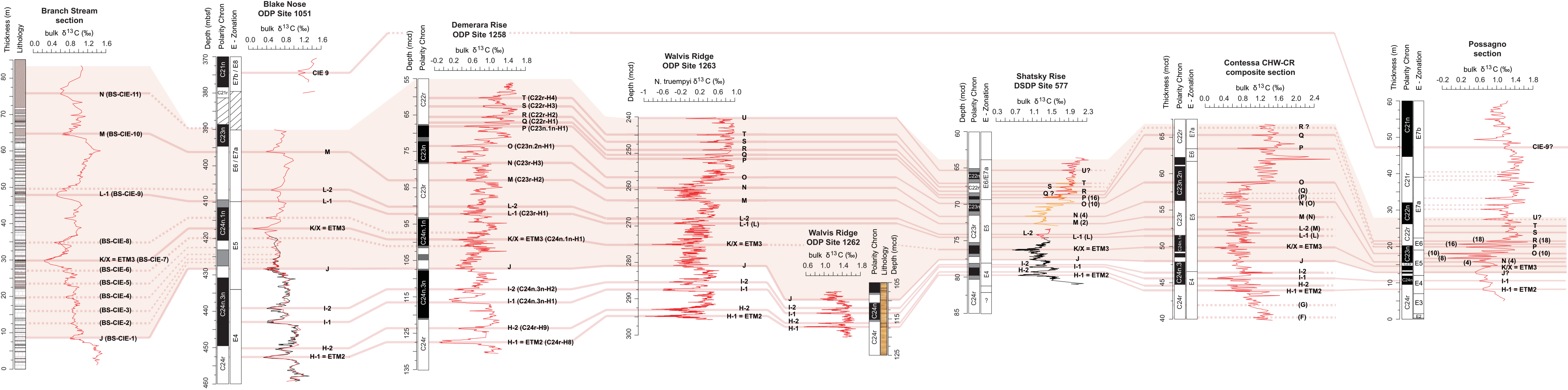

Supplement: Supplementary file 2 — Figure S1 [file PALO-32-1115-s001.pdf]

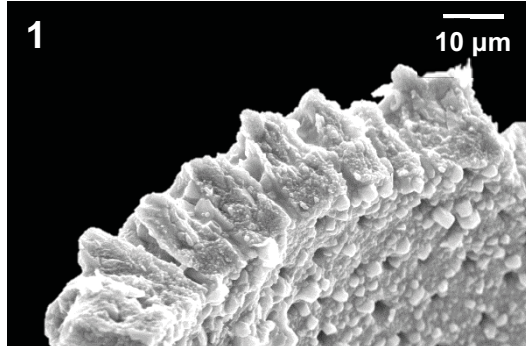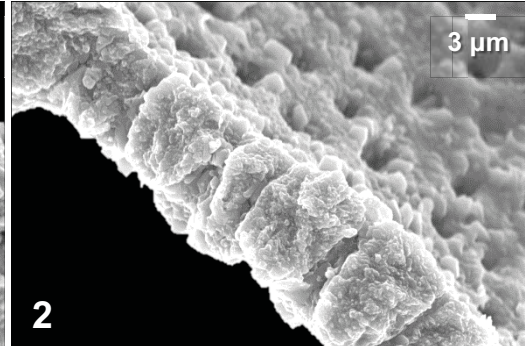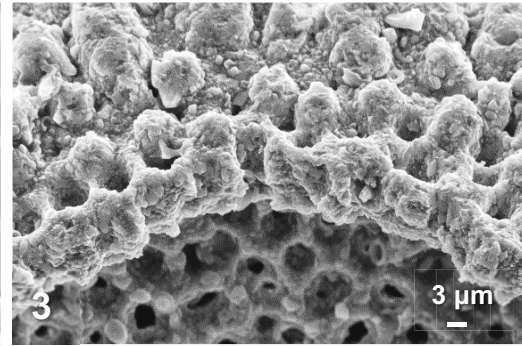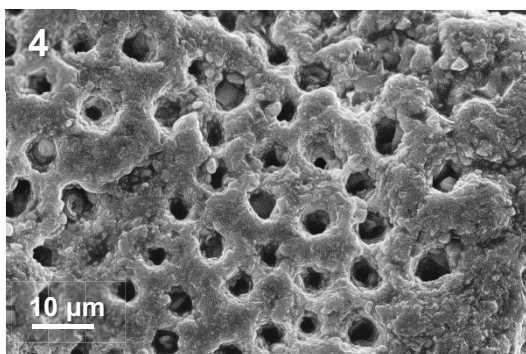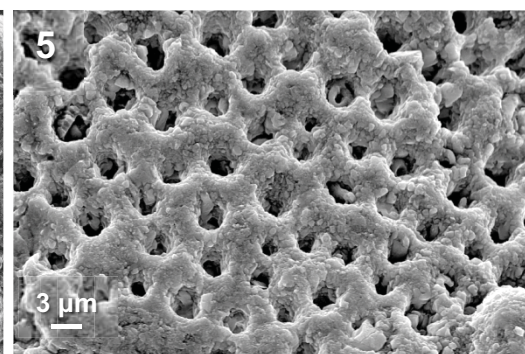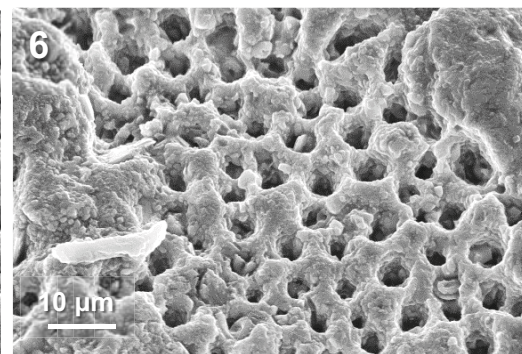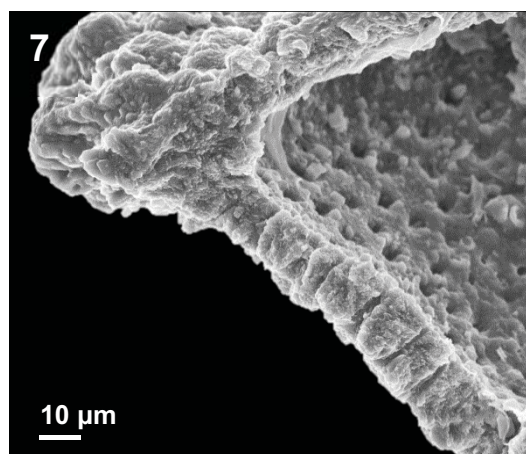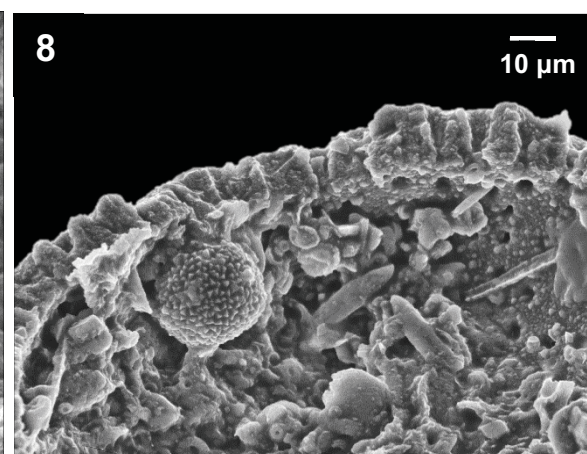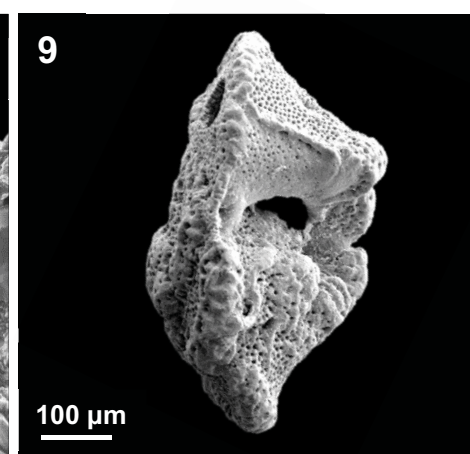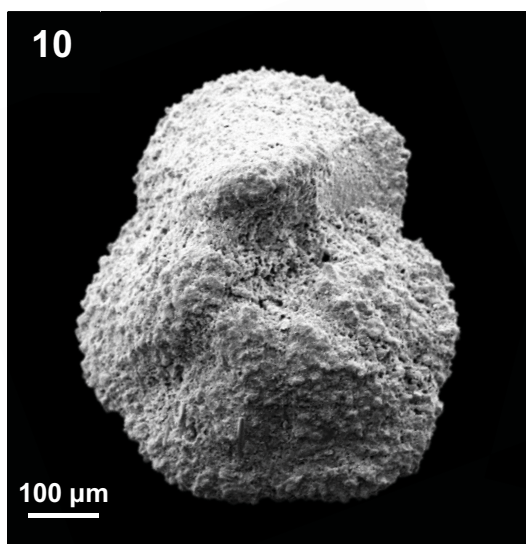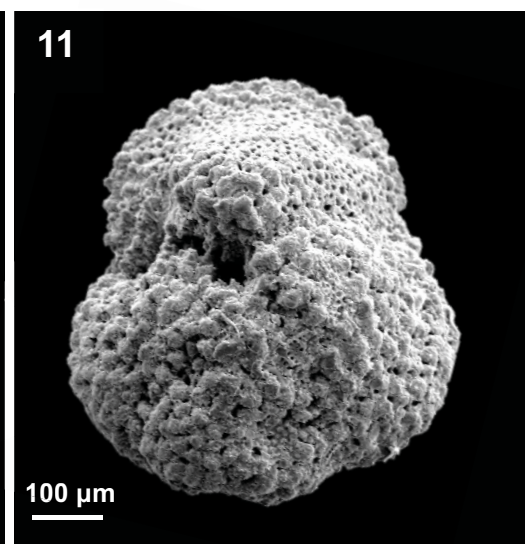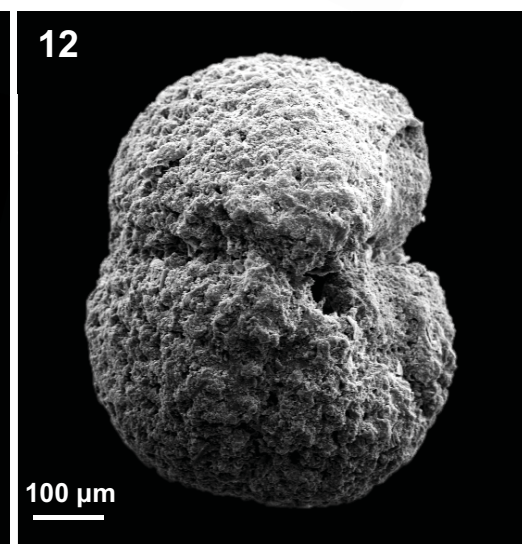

Supplement: Supplementary file 3 — Figure S2 [file PALO-32-1115-s002.pdf]
