# Supplementary material for: Did Photosymbiont Bleaching Lead to the Demise of Planktic Foraminifer Morozovella at the Early Eocene Climatic Optimum?
Source: Paleoceanography. 2017 Nov 6;32(11):1115–36. doi: 10.1002/2017PA003138 (PMC5784393; doi:10.1002/2017PA003138)
Supplement: Supplementary file 4 — Table S1 [file PALO-32-1115-s004.pdf]

Table S1. Carbon and oxygen stable-isotope composition of early Eocene bulk carbonate at Site 1051.

Leg	Site	Hole	Core	Section	Interval		Volume	Depth (mbsf)	$\delta^{13}C$ (‰)	$\delta^{18}O$ (‰)
					Top Depth	Bottom Depth				
171	1051	A	40	1	20	22	20	369,9	–	–
171	1051	A	40	1	70	72	20	370,4	1,5	0,21
171	1051	A	40	1	120	122	20	370,9	1,41	-0,02
171	1051	A	40	2	20	22	20	371,4	1,38	0
171	1051	A	40	2	70	72	20	371,9	1,44	0,01
171	1051	A	40	2	122	124	20	372,42	1,41	0,06
171	1051	A	40	3	20	22	20	372,9	1,38	-0,01
171	1051	A	40	3	70	72	20	373,4	1,32	0,13
171	1051	A	40	3	120	122	20	373,9	1,09	0,03
171	1051	A	40	4	20	22	20	374,4	1,25	0,17
171	1051	A	40	4	70	72	20	374,9	1,1	-0,16
171	1051	A	40	4	120	122	20	375,4	1,21	-0,91
171	1051	A	40	5	20	22	20	375,9	1,25	-0,46
171	1051	A	40	5	70	72	20	376,4	1,33	-0,07
171	1051	A	40	cc	20	22	20	377	1,31	0,27
171	1051	A	41	1	20	24	20	379,5	1,46	-1,11
171	1051	A	41	1	70	72	20	380	1,2	-0,91
171	1051	A	42	1	20	22	20	390,1	0,57	-0,67
171	1051	A	42	1	70	72	20	390,6	0,83	-0,58
171	1051	A	42	1	120	122	20	391,1	0,72	-0,62
171	1051	A	42	2	20	22	20	391,6	0,79	-0,5
171	1051	A	42	2	70	72	20	392,1	0,77	-0,57
171	1051	A	42	2	120	122	20	392,6	0,71	-0,41
171	1051	A	42	3	20	22	20	393,1	0,74	-0,41
171	1051	A	42	3	70	72	20	393,6	0,72	-0,47
171	1051	A	42	3	120	122	20	394,1	0,61	-0,46
171	1051	A	42	4	20	22	20	394,6	0,68	-0,44
171	1051	A	42	4	70	72	20	395,1	0,56	-0,52
171	1051	A	42	4	125	127	20	395,65	0,55	-0,57
171	1051	A	42	5	20	22	20	396,1	0,39	-0,53
171	1051	A	42	5	70	72	20	396,6	0,8	-0,48
171	1051	A	42	5	120	122	20	397,1	0,77	-0,39
171	1051	A	42	6	25	27	20	397,65	0,87	-0,41
171	1051	A	42	6	70	72	20	398,1	0,97	-0,27
171	1051	A	42	6	120	122	20	398,6	0,99	-0,33
171	1051	A	42	7	20	22	20	399,1	0,91	-0,35
171	1051	A	43	1	22	24	20	399,72	0,81	-0,41
171	1051	A	43	1	70	72	20	400,2	0,78	-0,39
171	1051	A	43	1	122	124	20	400,72	0,9	-0,17
171	1051	A	43	2	20	22	20	401,2	0,86	-0,17
171	1051	A	43	2	68	70	20	401,68	0,84	-0,35
171	1051	A	43	2	120	122	20	402,2	0,82	-0,23
171	1051	A	43	3	20	22	20	402,7	0,81	-0,26
171	1051	A	43	3	69	71	20	403,19	0,77	-0,31
171	1051	A	43	3	118	120	20	403,68	0,79	-0,23
171	1051	A	43	4	18	21	20	404,18	0,8	-0,2
171	1051	A	43	4	70	72	20	404,7	0,77	-0,29
171	1051	A	43	4	122	124	20	405,22	0,75	-0,22
171	1051	A	43	5	20	22	20	405,7	0,71	-0,33
171	1051	A	43	5	68	70	20	406,18	0,61	-0,54
171	1051	A	43	5	120	122	20	406,7	0,5	-0,56
171	1051	A	43	6	17	19	20	407,17	0,7	-0,37
171	1051	A	43	6	68	70	20	407,68	0,71	-0,37
171	1051	A	43	6	122	124	20	408,22	0,69	-0,02
171	1051	A	43	7	18	20	20	408,68	0,63	-0,59
171	1051	A	44	1	20	22	20	409,3	0,67	-0,46
171	1051	A	44	1	70	72	20	409,8	0,66	-0,43
171	1051	A	44	1	120	122	20	410,3	0,83	-0,27
171	1051	A	44	2	20	22	20	410,8	0,78	-0,47
171	1051	A	44	2	70	72	20	411,3	0,79	-0,19
171	1051	A	44	2	120	122	20	411,8	0,67	-0,46
171	1051	A	44	3	20	22	20	412,3	0,77	-0,37
171	1051	A	44	3	70	72	20	412,8	0,7	-0,3
171	1051	A	44	3	120	122	20	413,3	0,62	-0,33

171	1051	A	44	4	20	22	20	413,8	0,63	-0,45
171	1051	A	44	4	70	72	20	414,3	0,39	-0,44
171	1051	A	44	4	120	122	20	414,8	0,7	-0,38
171	1051	A	44	5	18	20	20	415,28	0,71	-0,38
171	1051	A	44	5	68	70	20	415,78	0,63	-0,32
171	1051	A	44	5	118	120	20	416,28	0,5	-0,32
171	1051	A	44	6	20	22	20	416,8	0,43	-0,38
171	1051	A	44	6	70	72	20	417,3	0,1	-0,82
171	1051	A	44	6	120	122	20	417,8	0,63	-0,42
171	1051	A	44	7	20	22	20	418,3	0,36	-0,68
171	1051	A	45	1	20	22	20	418,9	0,41	-0,56
171	1051	A	45	1	70	72	20	419,4	0,48	-0,75
171	1051	A	45	1	120	122	20	419,9	0,41	-0,82
171	1051	A	45	2	20	22	20	420,4	0,58	-0,49
171	1051	A	45	2	69	71	20	420,89	0,72	-0,51
171	1051	A	45	2	120	122	20	421,4	0,63	-0,6
171	1051	A	45	3	20	22	20	421,9	0,65	-0,89
171	1051	A	45	3	70	72	20	422,4	0,51	-0,74
171	1051	A	45	3	120	122	20	422,9	0,62	-0,48
171	1051	A	45	4	3	5	30	423,23	0,59	-0,61
171	1051	A	45	4	22	24	20	423,42	0,65	-0,43
171	1051	A	45	4	47	49	30	423,67	0,65	-0,42
171	1051	A	45	4	68	70	20	423,88	0,7	-0,29
171	1051	A	45	4	98	100	30	424,18	0,61	-0,6
171	1051	A	45	4	120	122	20	424,4	0,59	-0,74
171	1051	A	45	5	1	3	30	424,71	0,69	-0,51
171	1051	A	45	5	18	20	20	424,88	0,7	-0,37
171	1051	A	45	5	48	50	30	425,18	0,64	-0,47
171	1051	A	45	5	70	72	20	425,4	0,63	-0,48
171	1051	A	45	5	72	74	30	425,42	0,61	-0,63
171	1051	A	45	5	89	91	30	425,59	0,62	-0,41
171	1051	A	45	5	109	111	30	425,79	0,54	-0,37
171	1051	A	45	5	120	122	20	425,9	0,61	-0,35
171	1051	A	45	5	128	130	30	425,98	0,61	-0,39
171	1051	A	45	5	138	140	30	426,08	0,58	-0,3
171	1051	A	45	6	4	6	30	426,24	0,58	-0,47
171	1051	A	45	6	9	11	30	426,29	0,62	-0,18
171	1051	A	45	6	18	20	30	426,38	0,64	-0,19
171	1051	A	45	6	20	22	20	426,4	0,62	-0,38
171	1051	A	45	6	29	31	30	426,49	0,58	-0,29
171	1051	A	45	6	39	41	30	426,59	0,56	-0,29
171	1051	A	45	6	47	49	30	426,67	0,55	-0,46
171	1051	A	45	6	61	63	30	426,81	0,58	-0,43
171	1051	A	45	6	71	73	20	426,91	0,54	-0,52
171	1051	A	45	6	73	75	30	426,93	0,58	-0,45
171	1051	A	45	6	80	82	30	427	0,59	-0,27
171	1051	A	45	6	120	122	20	427,4	0,66	-0,2
171	1051	A	45	6	84	86	30	427,04	0,56	-0,34
171	1051	A	45	6	89	91	30	427,09	0,58	-0,43
171	1051	A	45	6	94	96	30	427,14	0,54	-0,34
171	1051	A	45	6	99	101	30	427,19	0,62	-0,27
171	1051	A	45	6	104	106	30	427,24	0,62	-0,36
171	1051	A	45	6	107	109	30	427,27	0,61	-0,35
171	1051	A	45	6	114	116	30	427,34	0,61	-0,44
171	1051	A	45	6	118	120	30	427,38	0,63	-0,37
171	1051	A	45	6	124	126	30	427,44	0,67	-0,31
171	1051	A	45	6	128	130	30	427,48	0,63	-0,38
171	1051	A	45	6	134	136	30	427,54	0,64	-0,42
171	1051	A	45	6	141	143	30	427,61	0,67	-0,46
171	1051	A	45	6	144	146	30	427,64	0,64	-0,36
171	1051	A	45	7	0	2	30	427,7	0,66	-0,28
171	1051	A	45	7	6	8	30	427,76	0,67	-0,45
171	1051	A	45	7	9	11	30	427,79	0,66	-0,28
171	1051	A	45	7	14	16	30	427,84	0,61	-0,48
171	1051	A	45	7	19	21	30	427,89	0,58	-0,53
171	1051	A	45	7	21	23	20	427,91	0,6	-0,47
171	1051	A	45	7	26	28	30	427,96	0,55	-0,49
171	1051	A	45	7	29	31	30	427,99	0,42	-0,6
171	1051	A	45	7	36	38	30	428,06	0,42	-0,69

171	1051	A	45	7	38	40	30	428,08	0,48	-0,69
171	1051	A	45	7	45	47	30	428,15	0,58	-0,59
171	1051	A	45	8	2	5	30	428,19	0,59	-0,47
171	1051	A	45	8	7	10	30	428,24	0,61	-0,45
171	1051	A	46	1	4	6	30	428,34	0,33	-0,83
171	1051	A	46	1	10	12	30	428,4	0,42	-0,92
171	1051	A	46	1	14	16	30	428,44	0,41	-0,92
171	1051	A	46	1	18	20	30	428,48	0,51	-0,72
171	1051	A	46	1	20	22	20	428,5	0,55	-0,44
171	1051	A	46	1	22	24	30	428,52	0,54	-0,74
171	1051	A	46	1	29	31	30	428,59	0,49	-0,67
171	1051	A	46	1	34	36	30	428,64	0,49	-0,78
171	1051	A	46	1	38	40	30	428,68	0,48	-0,94
171	1051	A	46	1	44	46	30	428,74	0,58	-0,77
171	1051	A	46	1	48	50	30	428,78	0,58	-0,71
171	1051	A	46	1	54	56	30	428,84	0,65	-0,51
171	1051	A	46	1	58	60	30	428,88	0,65	-0,37
171	1051	A	46	1	64	66	30	428,94	0,63	-0,54
171	1051	A	46	1	68	70	30	428,98	0,59	-0,35
171	1051	A	46	1	71	73	20	429,01	0,65	-0,14
171	1051	A	46	1	77	79	30	429,07	0,69	-0,3
171	1051	A	46	1	89	91	30	429,19	0,56	-0,55
171	1051	A	46	1	98	100	30	429,28	0,64	-0,55
171	1051	A	46	1	108	110	30	429,38	0,71	-0,49
171	1051	A	46	1	119	121	30	429,49	0,82	-0,17
171	1051	A	46	1	120	122	20	429,5	0,75	-0,43
171	1051	A	46	1	129	131	30	429,59	0,82	-0,24
171	1051	A	46	1	140	142	30	429,7	0,82	-0,3
171	1051	A	46	2	1	3	30	429,81	0,76	-0,6
171	1051	A	46	2	9	11	30	429,89	0,72	-0,56
171	1051	A	46	2	20	22	20	430	0,76	-0,47
171	1051	A	46	2	22	24	30	430,02	0,86	-0,75
171	1051	A	46	2	36	38	30	430,16	0,74	-0,53
171	1051	A	46	2	57	59	30	430,37	0,78	-0,69
171	1051	A	46	2	70	72	20	430,5	0,76	-0,48
171	1051	A	46	2	98	100	30	430,78	0,84	-0,56
171	1051	A	46	2	120	122	20	431	0,88	-0,35
171	1051	A	46	3	1	3	30	431,31	0,91	-0,45
171	1051	A	46	3	22	24	20	431,52	0,9	-0,43
171	1051	A	46	3	48	50	30	431,78	0,8	-0,51
171	1051	A	46	3	68	70	20	431,98	0,82	-0,47
171	1051	A	46	3	97	99	30	432,27	0,79	-0,7
171	1051	A	46	3	120	122	20	432,5	0,8	-0,47
171	1051	A	46	4	2	4	30	432,82	0,9	-0,51
171	1051	A	46	4	18	20	20	432,98	0,77	-0,48
171	1051	A	46	4	70	72	20	433,5	0,81	-0,44
171	1051	A	46	4	118	120	20	433,98	0,89	-0,48
171	1051	A	46	5	18	20	20	434,48	0,88	-0,39
171	1051	A	46	5	74	76	20	435,04	0,91	-0,39
171	1051	A	46	5	120	122	20	435,5	0,86	-0,34
171	1051	A	46	6	18	20	20	435,98	0,77	-0,42
171	1051	A	46	6	70	72	20	436,5	0,79	-0,39
171	1051	A	46	6	120	122	20	437	0,78	-0,45
171	1051	A	46	7	22	24	20	437,52	0,89	-0,36
171	1051	A	47	1	18	20	20	438,08	0,75	-0,52
171	1051	A	47	1	74	76	20	438,64	0,65	-0,51
171	1051	A	47	1	118	120	20	439,08	0,48	-0,6
171	1051	A	47	2	17,5	19,5	20	439,575	0,77	-0,38
171	1051	A	47	2	72	74	20	440,12	0,76	-0,41
171	1051	A	47	2	117,5	119,5	20	440,575	0,79	-0,55
171	1051	A	47	3	18	20	20	441,08	0,9	-0,37
171	1051	A	47	3	68	70	20	441,58	0,8	-0,57
171	1051	A	47	3	123	125	20	442,13	0,77	-0,4
171	1051	A	47	4	15,5	17,5	20	442,555	0,53	-0,67
171	1051	A	47	4	73	75	20	443,13	0,48	-0,79
171	1051	A	47	4	118	120	20	443,58	0,91	-0,51
171	1051	A	47	5	23	25	20	444,13	1,06	-0,39
171	1051	A	47	5	68	70	20	444,58	1,09	-0,6
171	1051	A	47	5	117,5	119,5	20	445,075	1,15	-0,61

171	1051	A	47	6	17	19	20	445,57	1,01	-0,5
171	1051	A	47	6	73	75	20	446,13	1,04	-0,65
171	1051	A	47	6	114	116	20	446,54	1,09	-0,49
171	1051	A	47	7	7	9	20	446,97	1,03	-0,47
171	1051	A	48	1	23	25	20	447,73	0,97	-0,52
171	1051	A	48	1	68	70	20	448,18	0,96	-0,74
171	1051	A	48	1	118	120	20	448,68	1,01	-0,72
171	1051	A	48	2	22	24	20	449,22	1,11	-0,45
171	1051	A	48	2	69	72	20	449,69	0,98	-0,64
171	1051	A	48	2	117	119	20	450,17	0,76	-0,81
171	1051	A	48	3	17	19	20	450,67	0,71	-0,43
171	1051	A	48	3	69	73	20	451,19	0,88	-0,36
171	1051	A	48	3	118	120	20	451,68	0,89	-0,3
171	1051	A	48	4	24	26	20	452,24	0,51	-0,67
171	1051	A	48	4	73	75	20	452,73	0,37	-0,73
171	1051	A	48	4	118	120	20	453,18	0,9	-0,43
171	1051	A	48	5	18	20	20	453,68	1,01	-0,28
171	1051	A	48	5	68	70	20	454,18	1,09	-0,54
171	1051	A	48	5	120	124	20	454,7	1,04	-0,35
171	1051	A	48	6	18	20	20	455,18	0,95	-0,22
171	1051	A	48	6	69	73	20	455,69	0,91	-0,56
171	1051	A	48	7	23	25	20	456,25	0,95	-0,14
171	1051	A	48	1	18	20	20	457,28	1,17	-0,42
171	1051	A	49	1	66	70	20	457,76	1,21	-0,7
171	1051	A	49	1	118	120	20	458,28	0,91	-0,87
171	1051	A	49	2	16	20	20	458,76	1,13	-0,09
171	1051	A	49	2	66	68	20	459,26	0,84	-0,9
