# Supplementary material for: Did Photosymbiont Bleaching Lead to the Demise of Planktic Foraminifer Morozovella at the Early Eocene Climatic Optimum?
Source: Paleoceanography. 2017 Nov 6;32(11):1115–36. doi: 10.1002/2017PA003138 (PMC5784393; doi:10.1002/2017PA003138)
Supplement: Supplementary file 7 — Table S4 [file PALO-32-1115-s007.pdf]

Table S3. Relative abundances (%) of Morozovella species at Site 1051 with respect to the total foraminferal population.

Hole	Core	Section	Interval (cm ±1)	Sample Depth (mbsf)	No. Total counted specimens	N. *Morozovella*	*Morozovella* (%)	*M.aequa* (%)
1051A	40	1	21	369,9	335	11	3	0
1051A	40	1	121	370,9	311	27	7,4	0
1051A	40	3	121	373,9	303	21	5,5	0
1051A	40	5	71	376,4	314	10	2,6	0
1051A	40	cc	21	377	338	40	10,5	0
1051A	41	1	21	379,5	301	37	9,8	0
1051A	42	1	21	390,1	219	20	6	0
1051A	42	3	121	394,1	211	24	6,2	0,3
1051A	42	6	26	397,65	385	39	9,8	0
1051A	43	2	69	401,68	285	43	10,8	0
1051A	43	4	19	404,18	295	34	8,5	0
1051A	43	5	69	406,18	336	29	7,1	0
1051A	44	1	21	409,3	310	72	17,5	0,2
1051A	44	1	71	409,8	315	59	14,5	0,5
1051A	44	3	21	412,3	332	49	12	0,5
1051A	44	4	21	413,8	290	27	13,2	0
1051A	44	5	69	415,78	327	28	8,5	0,9
1051A	44	6	121	417,8	375	53	14,1	0
1051A	45	1	121	419,9	371	63	16,9	2,2
1051A	45	3	21	421,9	389	29	7,8	1
1051A	45	3	71	422,4	352	78	22	1,1
1051A	45	3	121	422,9	186	22	11,8	0
1051A	45	4	23	423,42	350	53	15,1	2,9
1051A	45	4	68	423,88	349	34	9,7	0
1051A	45	4	120	424,4	330	69	20,9	0
1051A	45	5	18	424,88	343	67	19,5	0,3
1051A	45	5	71	425,4	307	55	18	0,3
1051A	45	6	72	426,9	333	65	19,5	1,5
1051A	45	6	121	427,4	263	35	13,3	0,4
1051A	45	7	22	427,91	250	37	14,8	1,2
1051A	45	7	30	427,99	331	51	15,4	0
1051A	45	7	37	428,06	347	39	11,2	0,9
1051A	45	7	39	428,08	342	50	14,6	0,6
1051A	45	7	46	428,15	348	54	15,5	0,3
1051A	45	8	3	428,19	384	64	16,7	0
1051A	45	8	8	428,24	359	45	12,5	0,3
1051A	46	1	5	428,34	325	95	29,2	1,2
1051A	46	1	11	428,4	340	95	27,9	0,3
1051A	46	1	15	428,44	329	113	34,3	1,2
1051A	46	1	19	428,48	336	124	36,9	0,6
1051A	46	1	21	428,5	245	111	53,5	0
1051A	46	2	21	430	315	130	54	1
1051A	46	2	71	430,5	242	101	42	1,2
1051A	46	4	71	433,5	248	95	38,3	0
1051A	46	4	119	433,98	247	98	43	2,5
1051A	46	5	19	434,48	223	85	39	2,3
1051A	47	2	18,5	439,57	293	132	45	3,5
1051A	47	3	69	441,58	282	118	41,8	4,6
1051A	47	5	24	444,13	341	132	38,7	2,1
1051A	48	2	70	449,69	322	101	31,4	3,1
1051A	48	4	25	452,24	259	138	46,3	5,1
